# Oxygen Vacancy-Induced Phase Transformations of Iron-Doped Titanium Oxide Nanostructures

**DOI:** 10.1021/acsnano.5c08093

**Published:** 2025-08-21

**Authors:** Guilherme B. Strapasson, Adrián S. Arjona, Joseph E. McPeak, Olivia Aalling-Frederiksen, Adam F. Sapnik, Nanna L. Baun, Heloisa N. Bordallo, Cristiane B. Rodella, Daniela Zanchet, Kirsten M. Ø. Jensen

**Affiliations:** † Institute of Chemistry, 344102 Universidade Estadual de Campinas, Campinas, São Paulo 13083-970, Brazil; ‡ Brazilian Synchrotron Light Laboratory, CNPEM, Campinas, São Paulo 13083-100, Brazil; § Department of Chemistry and Nano-Science Center, 4321 University of Copenhagen, Universitetsparken 5, Copenhagen Ø 2100, Denmark; ∥ Niels Bohr Institute, University of Copenhagen, Universitetsparken 5, Copenhagen Ø 2100, Denmark

**Keywords:** nanomaterials, reducible metal oxides, oxygen vacancies, pair distribution function, defect engineering

## Abstract

Oxygen vacancies play a pivotal role in tailoring the electronic, optical, and catalytic properties of reducible metal oxides. Here, we provide a complete overview of oxygen vacancy-induced structural evolution of iron-doped titanium oxide nanomaterials with insights into their synthesis, formation, and crystallization processes. Structural analysis combining multiple techniques reveals the formation of anatase nanoparticles at low Fe loadings (i.e., ≤10 at. % Fe). At intermediate Fe concentrations (i.e., 15–20 at. % Fe), a mixture of anatase and rutile forms with the presence of extended disordered defects similar to crystallographic shear planes. These become more notable at high Fe loadings (i.e., ≥30 at. % Fe) with the complete transition to the rutile phase with a high density of defects. Moreover, we provide important information on the nucleation, growth, and crystallization processes during synthesis, emphasizing the impact of Fe atom incorporation on the TiO_2_ lattice, the formation of reaction intermediates, and the structural evolution at the nano regime. The ability to control oxygen vacancies and engineer defects in Fe-doped TiO_2_ allows for the optimization of charge transport, enhancing catalytic activity and tuning optical properties for applications in environmental remediation, sensing, and next-generation semiconductor technologies.

## Graphical abstract

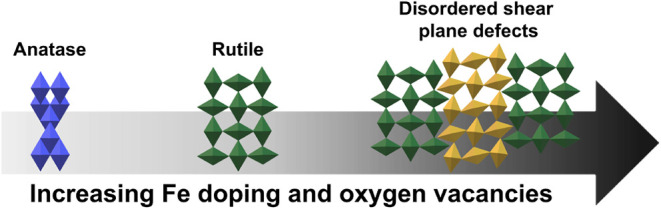

## Introduction

Intrinsic or extrinsic oxygen vacancies are the most common defects in reducible metal oxides. These oxygen vacancies can influence electronic and structural properties, chemical reactivity, and oxygen mobility, and thus play a key role in diverse research fields, such as catalysis, sensors, and energy conversion and storage.^1–5^ At the nanoscale, size-induced structural changes in the atomic structure of metal oxides can take place, favoring the generation of defective, distorted, and amorphous materials,^6–8^ facilitating the generation of oxygen vacancies. The engineering of oxygen vacancies and their relationship with the structural features of nanostructured metal oxides is essential for tailoring the properties of functional materials, e.g., enhancing catalytic performance and charge transport.

Titanium oxide (TiO_2_) is among the most studied reducible metal oxides due to its chemical stability, redox properties, and high availability.^9^ TiO_2_ can be found in three main crystalline polymorphs (i.e., rutile, anatase, and brookite; Scheme 1a–c), each distinguished by unique arrangements of the [TiO_6_] octahedra (i.e., edge- or corner-sharing). The structural features of the different polymorphs can dictate the formation and stabilization of the oxygen vacancies. For example, density functional theory (DFT) studies have shown that, at high concentrations of oxygen vacancies, rutile TiO_2_ is more thermodynamically stable than anatase. This stabilization is attributed to the greater structural flexibility associated with the lower number of edge-sharing octahedra in the rutile polymorph.^10^ Oxygen vacancies can also influence the stability of polymorphs, favoring the anatase-to-rutile phase transition, and/or the formation of Wadsley and Magnéli extended defects.^10–12^ Wadsley defects emerge from the ordering and subsequent collapse of oxygen vacancies into two-dimensional crystallographic shear planes (i.e., two-dimensional extended defects).^13,14^ As the concentration of these defects increases, they periodically stack, leading to the formation of Magnéli phases (i.e., Ti_*n*_O_2*n*–1_, three-dimensional extended defects).^15–18^ The Magnéli structure is derived from the rutile TiO_2_ lattice, where every *n*th layer exhibits oxygen deficiency, giving rise to periodic crystallographic shear planes (Scheme 1d). Within these shear planes, the [TiO_6_] octahedra adopt face-sharing configurations to accommodate the oxygen deficiency caused by the formation of oxygen vacancies.^19^

**Scheme 1:**
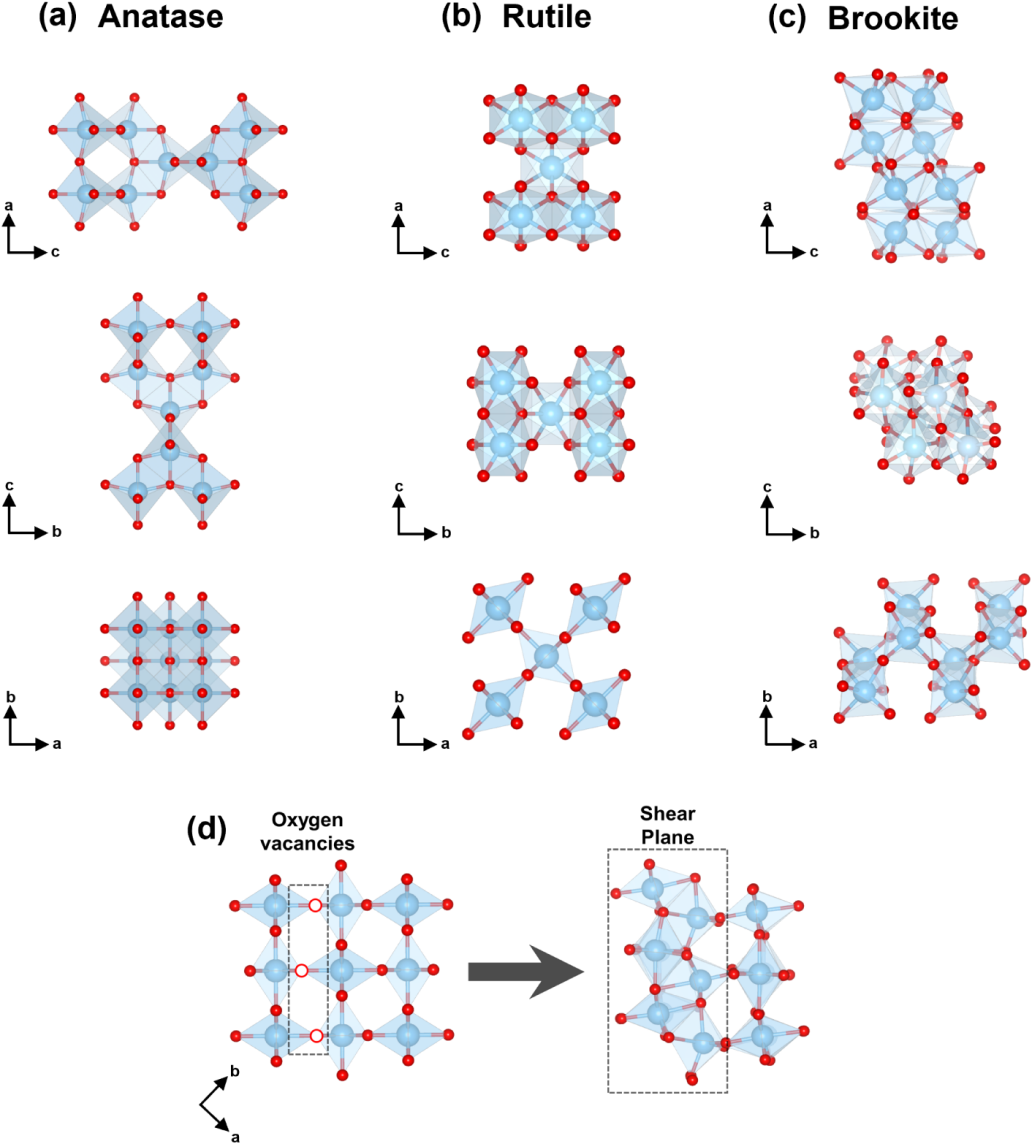
Structural Representations of (a) Anatase, (b) Rutile, and (c) Brookite Unit Cells; and (d) Ordered Oxygen Vacancies Within the Rutile Structure (Left Representation, Highlighted by the Dashed Rectangle) Inducing the Formation of Shear Planes (Right Representation, Highlighted by the Dashed Rectangle). Full Red Circles Represent Oxygen, Open Red Circles Represent Oxygen Vacancies, and Full Blue Circles Represent Titanium

Strategies such as aliovalent doping, thermal treatment under variable atmospheres, and high-energy particle bombardment enable precise control of vacancy concentration.^20–23^ Adding aliovalent dopants into the metal oxide structure can modify its redox properties by the incorporation of strain and structural distortions, increasing the oxygen vacancy concentration.^22^ A notable example is the addition of Fe atoms into the TiO_2_ lattice, where the substitution of Ti^4+^ by Fe^3+^ introduces an effective charge deficiency, promoting the formation of oxygen vacancies as a charge compensation mechanism to maintain overall charge neutrality. Additionally, the difference in ionic radii between Fe^3+^ (65 pm) and Ti^4+^ (61 pm) induces lattice distortions, further facilitating oxygen vacancy formation and modifying the material reducibility.^21,24,25^ Previous reports have demonstrated that Fe-doping can trigger the anatase-to-rutile phase transition,^26^ consistent with the structural influence of oxygen vacancy generation. However, at Fe loadings greater than ca. 10 at. %, partial phase segregation into an iron oxide phase is known to take place, hindering the total incorporation of Fe atoms into the TiO_2_ lattice.^27,28^ Moreover, the formation of the Fe_2_TiO_5_ pseudobrookite phase can be promoted at high annealing temperatures (i.e., ≥700 °C).^27,29^

The challenge of incorporating Fe loadings above 10 at. % into the TiO_2_ lattice without triggering phase segregation hinders mapping any systematic correlation between oxygen vacancy formation and the structural evolution of Fe-doped TiO_2_ materials. However, understanding the mechanisms underlying oxygen vacancy-induced phase transformations is crucial for the rational design and engineering of defects in Fe-doped titanium oxides, which are valuable for enhancing their optical, electronic, and catalytic properties. At the nanoscale, the solid solution solubility limits can be extended due to surface energy effects, which stabilize higher dopant concentrations and suppress phase segregation.^30–32^ The decreased surface energy can accommodate greater defect concentrations, including oxygen vacancies, compensating for charge imbalances introduced by Fe doping. These strategies have been employed in this work to extend the solid solution saturation limit of Fe-doped TiO_2_ systems.

Concerning applications, Fe-doped TiO_2_ nanomaterials have attracted increasing attention due to their defect-driven functional properties, which are directly linked to the formation of oxygen vacancies and associated structural modifications. In photocatalytic applications, Fe doping enhances visible-light absorption and promotes the generation of oxygen vacancies, which contribute to improved charge separation and catalytic activity under solar irradiation.^33,34^ In addition to photocatalysis, Fe-doped TiO_2_ nanomaterials have been explored in energy storage systems, where defect-rich structures enhance the ion transport and storage capabilities. In Na-ion batteries, Fe doping increases the density of active sites and promotes the formation of oxygen vacancies, which facilitate Na^+^ diffusion and contribute to improved storage capacity and cycling stability.^35,36^ Similarly, in Mg-ion batteries, Fe doping introduces oxygen vacancies that create favorable adsorption sites and improve Mg^2+^ mobility, enabling surface-dominated storage mechanisms and faster diffusion kinetics compared to undoped TiO_2_.^37^ These advances reinforce the importance of controlling oxygen vacancies and defects in order to tailor the properties of Fe-doped TiO_2_ nanomaterials for targeted applications.

This work elucidates the structural evolution of Fe-doped TiO_2_ nanostructures, overcoming phase segregation limitations even at Fe loadings up to 50 at. %. We have established key correlations between Fe loading, oxygen vacancies, phase transformations, and defect formation. The nucleation, growth, and crystallization processes were also investigated, offering a deeper understanding of the formation and structural evolution of Fe-doped nanomaterials as a function of synthesis time. Our main findings reveal that, upon incorporation of Fe loadings ≥30 at. %, extensive defects appear in the rutile structure, which we describe as disordered shear planes. These shear planes play a crucial role in stabilizing the crystal lattice under high oxygen vacancy concentrations, directly linking structural transformations to defect formation.

## Results and Discussion

### Structural Modifications of Fe-Doped TiO_2_

The Fe-doped TiO_2_ nanostructures were synthesized via a hydrothermal method utilizing water-soluble metal complexes as synthesis precursors.^38,39^ Metal complexes facilitate solid solution formation by providing a controlled coordination environment, where ligands dictate the spatial arrangement and distribution of metal cations, promoting homogeneous incorporation into the lattice. Here, we explored synthesis employing up to 50 at. % Fe loadings (i.e., Ti_1–*x*_Fe_*x*_O_2–0.5*x*_, 0 ≤ *x* ≤ 0.5, synthesis time of 20 h), aiming to evaluate the oxygen vacancy generation upon Fe incorporation into the TiO_2_ lattice. Inductively coupled plasma optical emission spectroscopy (ICP-OES) measurements demonstrated that Fe incorporation was close to the nominal values up to 15 at. % Fe; however, at higher loadings, partial incorporation of ca. 70% of the nominal loading occurred (Figure S1 and Table S1).

Powder X-ray diffraction (PXRD) analysis showed that undoped TiO_2_ crystallizes in the anatase phase (Figure 1a), with crystallite domains of ca. 10 nm (obtained by Le Bail refinement). The incorporation of Fe during synthesis led to smaller mean anatase crystallite domain sizes (from ca. 10 to 7 nm) and a slight expansion of the unit cell up to ca. 0.15% (Figure S2a). Fe loadings greater than 10 at. % led to the coexistence of anatase and rutile, while values greater than 20 at. % induced the formation of single-phase rutile nanoparticles. Similarly, a decrease in the rutile mean crystallite domains (from ca. 19 to 9 nm) and an expansion of its unit cell (up to ca. 0.3%) occurred upon Fe loading increase (Figure S2b). Although phase transformations could be influenced by pH, we rule out such effects under our synthesis conditions, as the initial solution exhibited only slight pH variations (from 9.3 to 8.6 as a function of Fe loading). The expansion of anatase and rutile unit cell volumes is consistent with Fe^3+^ incorporation into the TiO_2_ lattice, as the ionic radius of Fe^3+^ (65 pm) is slightly larger than that of Ti^4+^ (61 pm).^40^ In both phases, the incorporation of Fe induced an increase in the a-lattice parameter and a decrease in the c-lattice parameter (Tables S2 and S3). Furthermore, Le Bail refinements indicate that the anatase structure described the samples well with Fe loading up to 10 at. % (Figure S3a–c). For Fe loadings ≥20 at. %, the positions of the reflections aligned with those of the rutile structure (Figure S3d–g); however, discrepancies in intensity suggest the influence of preferred orientation or preferential growth along the *c*-axis. Additionally, the refinements reveal features in the diffraction pattern at ca. 3.8 and 4.5 Å^–1^ that are not fully accounted for by the rutile structure.

**Figure 1:**
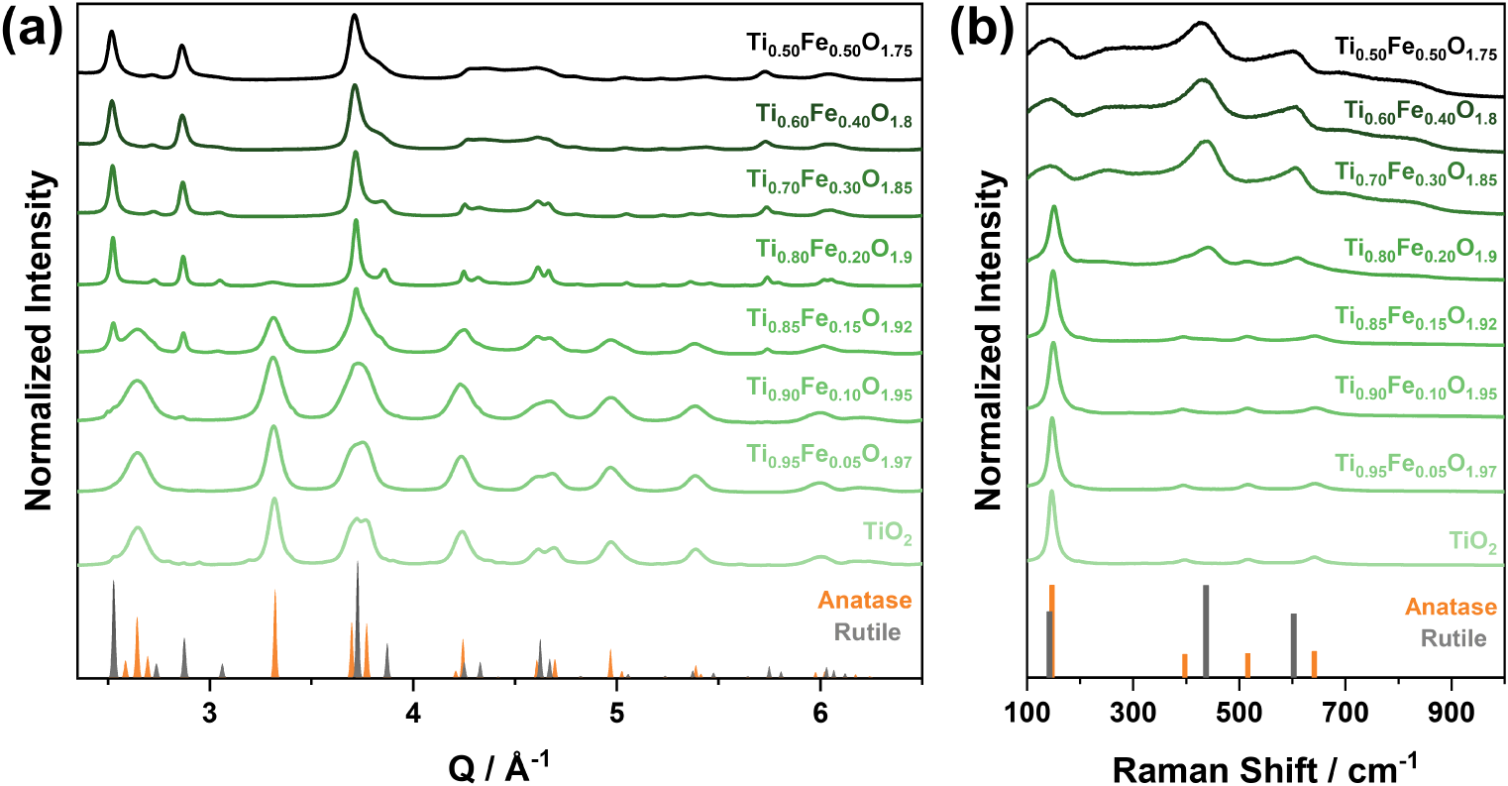
(a) Synchrotron PXRD and (b) Raman data of Ti_1–*x*_Fe_*x*_O_2–0.5*x*_ nanostructures as a function of the Fe loading (0 ≤ *x* ≤ 0.5).

Raman spectroscopy measurements complemented the X-ray scattering data (Figure 1b). The anatase phase (space group *I4*_1_ */amd*) shows major Raman bands at 146, 396, 515, and 640 cm^–1^, attributed to the Raman-active modes with the symmetries of *Eg*, *B1g*, (A1g, B1g), and *Eg*, respectively. The main Raman bands of the rutile phase (space group *P4*_2_ */mnm*) appear at 141 (superimposed with the 144 cm^–1^ *Eg* mode of the anatase phase), 436, and 602 cm^–1^, ascribed to the *B1g*, *Eg*, and *A1g* modes, respectively. As the Fe content increased (up to 20 at. %), the anatase Raman modes broadened (*Eg* mode FWHM from ca. 15 to 21 cm^–1^) and shifted to higher wavenumbers (*Eg* mode from ca. 146 to 151 cm^–1^, Figure S2c). These changes suggest a greater dispersion in metal–oxygen bond length and a decrease in their average value, respectively, consistent with Fe insertion into the TiO_2_ lattice and the generation of oxygen vacancies. Upon oxygen atom removal from the lattice, the nearest Ti atoms tend to relax away from the vacancy to stabilize the structure, causing the reduction of the metal–oxygen bond length.^20^

Raman measurements also demonstrated that the 15 at. % Fe (i.e., Ti_0.85_Fe_0.15_O_1.92_) sample did not follow the observed trends (Figure S2c). The data suggest that at the threshold where the anatase-to-rutile phase transition occurred, there was a depletion of Fe from the anatase phase and an enrichment of Fe in the rutile phase and/or leaching to the supernatant (Figure S1 and Table S1). The rutile Raman modes showed broadening (*Eg* mode FWHM from 45 to 66 cm^–1^) and shifting toward lower wavenumbers (*Eg* mode from 442 to 429 cm^–1^) upon Fe concentration increase (i.e., from 20 to 50 at. %, Figure S2d), respectively. We relate these effects in the spectra to a greater dispersion of the metal–oxygen bond length and an increase in its average value. Although the anatase samples followed the expected decrease in the average metal–oxygen bond length upon Fe incorporation (i.e., attributed to the presence of oxygen vacancies), we observe the opposite trend for rutile. This effect may be associated with the generation of more defective and distorted rutile crystalline domains arising from the high Fe loadings (and the associated increase in oxygen vacancies), in agreement with the notable broadening and asymmetry of the Raman modes (Figures 1b and S2d).

A more detailed structural analysis was conducted by employing X-ray total scattering and PDF analysis. Initially, real-space Rietveld refinements were performed in the short- to medium-range [1.5–20 Å] (Figures 2 and S4) employing models of anatase and rutile based on the phases identified by PXRD and Raman. As expected, undoped TiO_2_ and samples with low loadings of Fe (i.e., up to 20 at. % Fe) could be described well with the anatase structure or a combination of anatase and rutile (Rw ≤ 0.18, Figures 2a,b and S4a–e). In contrast, when fitting the PDFs from the samples with higher Fe loadings with the rutile structure, we observed a decrease in the goodness of the fits (0.26 ≤ Rw ≤ 0.38, Figures 2c, S4f–h and Table S4). The residual PDF of Fe loadings ≥20 at. % highlights that some features at *r* ≥ 9 Å evolved upon Fe increase (Figure 2d, highlighted in green), indicating Fe-induced structural changes that are not fully described by the rutile model. At lower *r*-values (*r* < 9 Å), the difference curve shows similar features, demonstrating that the local structure was relatively well described by a single rutile phase model. These findings suggest the generation of structural defects affecting the medium- to long-range order. Based on previous studies on defect formation in the TiO_2_ rutile system,^6^ such defects are likely associated with the collapse of a lattice enriched in oxygen vacancies into crystallographic shear planes as a way of stabilizing the crystal lattice. These shear planes promote the formation of extended defects associated with more reduced titanium oxide phases, commonly identified as Magnéli phases (i.e., Ti_*n*_O_2*n*–1_).^13,41–43^

**Figure 2:**
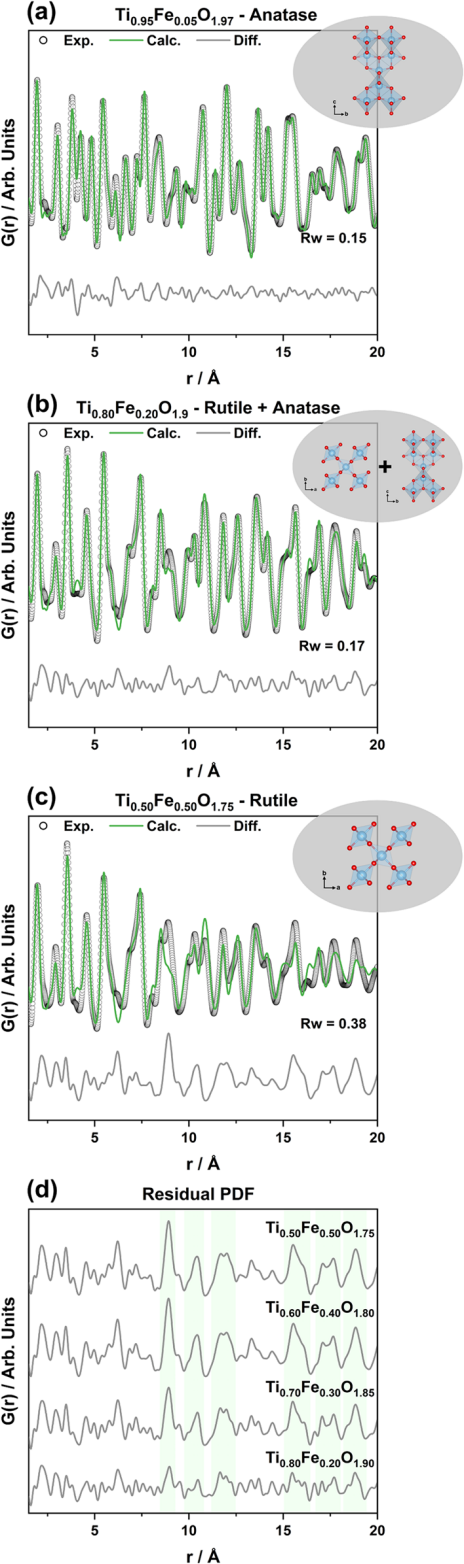
Short- to medium-range PDF refinements from *ex situ* measurements of (a) Ti_0.95_Fe_0.05_O_1.97_, (b) Ti_0.80_Fe_0.20_O_1.90_, and (c) Ti_0.50_Fe_0.50_O_1.75_; (d) residual PDF of refined Ti_1–*x*_Fe_*x*_O_2–0.5*x*_, 0.20 ≤ *x* ≤ 0.5 (refinements are shown in Figure S4). Anatase and rutile TiO_2_ polymorphs were used as models for the refinements. The phases employed for each refinement are shown at the top of the plots. The evolution of residual features as a function of the Fe at. % loading is highlighted in green.

To investigate this hypothesis further, and before doing additional PDF analysis, we took a closer look at the PXRD data. A pronounced evolution of the broadening and asymmetry of Bragg reflections can be seen at ca. 3.0, 3.8, and 4.5 Å^–1^ upon Fe loading increase (Figure S5a, highlighted in gray). Additionally, the PDFs show a clear evolution of the feature at ca. 9 Å with an increase in Fe loading (Figure S5b, highlighted in gray), associated with one of the main fingerprints observed in Figure 2d. By comparing the features in the experimental PXRD patterns with simulated data from multiple titanium oxide (Figure S6), iron oxide (Figure S7), and titanium–iron oxide (Figure S8) phases, we identified three possible candidates with a Magnéli-like structure: Ti_3_O_5_, Ti_5_O_9_, and TiFe_2_O_5_. While we did not observe either of these structures as distinct phases in the PXRD data, they may contain structural motifs that can aid in better describing the medium-range structure in the PDF. The possible candidates were therefore further evaluated by Pearson correlation analysis of their simulated PDFs compared to TiO_2_ polymorphs over different *r* regions (i.e., up to 5 Å, 20 Å, and 80 Å, Figure S9). This statistical approach (described in detail in the Supporting Information) evaluates the similarity between the simulated PDFs, with 1 representing a perfect match.^44^ The Pearson correlation results indicate that anatase has a low degree of similarity with the other phases in all *r* regions. On the other hand, rutile was highly correlated with the possible Magnéli-like phases (i.e., Ti_3_O_5_, Ti_5_O_9_, and TiFe_2_O_5_) in the short-range [*r* ≤ 5 Å] (Figure S9a), in line with the observations from the residual PDFs (Figure 2d), which show that the local structure can be described by rutile. As expected, a decrease in the degree of similarity occurred when *r* was increased to the medium-range [*r* ≤ 20 Å] and long-range [*r* ≤ 80 Å] (Figure S9b,c). Instead, Ti_5_O_9_ presented the highest correlation values when compared to rutile, representing 0.80 (short-range), 0.43 (medium-range), and 0.34 (long-range). Defects arising from the rutile structure remain embedded within it while still retaining rutile-like blocks, highlighting their intrinsic correlation. Based on these findings, we employed Ti_5_O_9_ as a second phase combined with rutile as a model to simulate the presence of structural defects. This approach should not be interpreted as the coexistence of two distinct phases but rather as a structural approximation in which Ti_5_O_9_ accounts for shear plane-like defects within the rutile structure. It provides a simple strategy to capture the medium-range order structure, and despite its simplicity, it offers a practical way to access structural motifs associated with the formation of such defects. This strategy aligns with previous studies in which PDF analysis proved essential for identifying structural motifs associated with defects and local distortions not detected by conventional diffraction methods.^6,45^

Focusing on the sample with the higher density of defects and worse fit against the rutile model (i.e., Ti_0.50_Fe_0.50_O_1.75_), PDF analysis of the short- to-medium range order [1.5–20 Å] was evaluated. By comparing the single-phase refinements employing rutile (Figure 3a) and Ti_5_O_9_ (Figure 3b) with a model combining both phases (Figure 3c), a clear improvement of the goodness of the fits was achieved from Rw of 0.38–0.43 to 0.28, respectively. This result demonstrates that including Ti_5_O_9_ in the refinement model to simulate the presence of shear plane-like defects improved the structural description of the sample. The same refinement strategy was expanded for the other Fe loadings (Figure S10), which also presented improvements in the goodness of the fits. The refined parameters for each of them are listed in Table S5. We emphasize that our refinement results should not be interpreted as two distinct phases (i.e., rutile and Ti_5_O_9_). Instead, we propose that the samples contain disordered extended defects resembling shear planes within the rutile structure. The structure of Ti_5_O_9_ is shown in Figure S11. It can be described as containing rutile-like blocks, along with shear planes, which are ordered in the structure. We hypothesize that the improvement of fit quality when including this phase appears as the samples contain motifs that are similar to the shear planes present in the Ti_5_O_9_ phase. They are, however, not crystallographically ordered and are thus not easily described using conventional Rietveld refinements. Therefore, although Rietveld refinement is valuable for long-range periodic systems, it is inherently limited in cases such as ours, where nonperiodic, defect-rich domains are dominant. In contrast, the two-phase PDF analysis makes it possible to describe the main structural motifs in the samples using a simple and small box model while still extracting significant structural information. It is important to note that our model represents a simplification that captures the dominant structural features but does not resolve the full complexity of the system.

**Figure 3:**
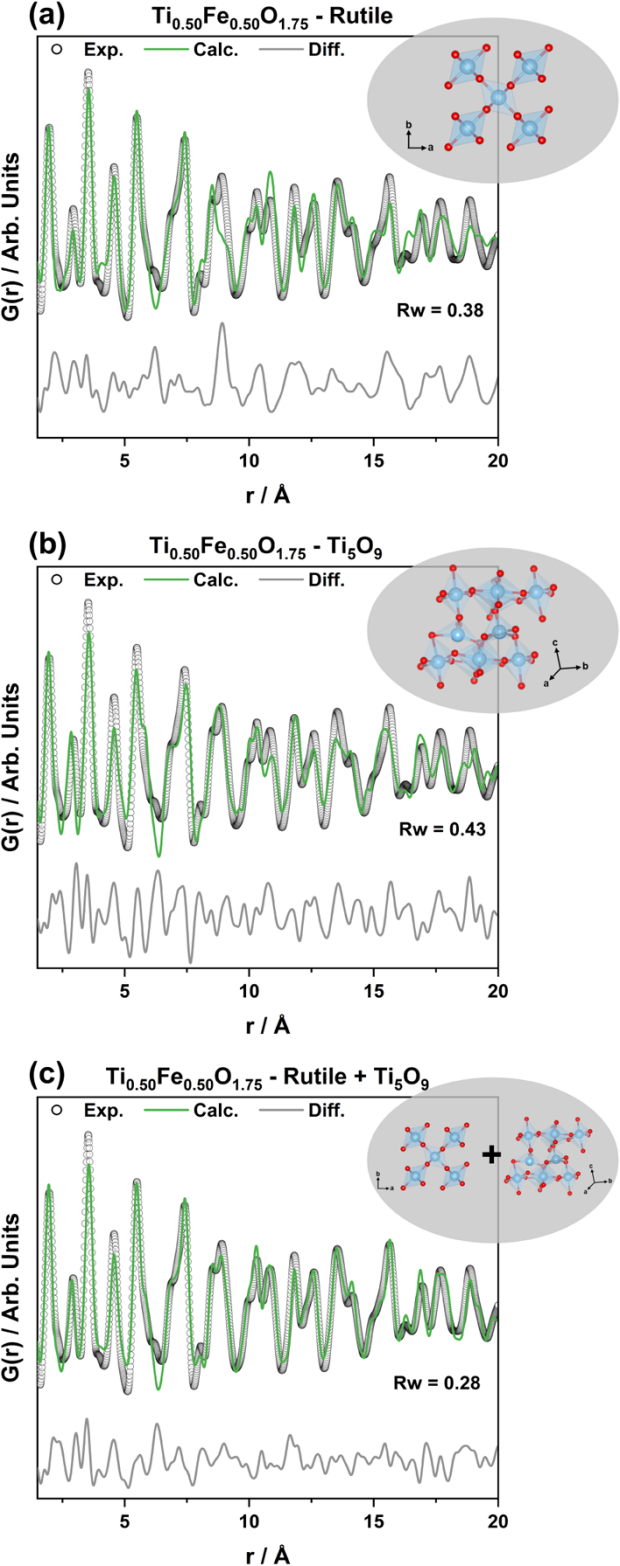
Short- to medium-range PDF refinements from *ex situ* measurements of Ti_0.50_Fe_0.50_O_1.75_ using (a) rutile, (b) Ti_5_O_9_, and (c) a combination of rutile + Ti_5_O_9_ as models. The phases employed for each refinement are shown at the top of the plots.

To better understand the spatial distribution of such defects, high-resolution scanning transmission electron microscopy (HR-STEM) was conducted. A representative image of Ti_0.50_Fe_0.50_O_1.75_ and the inverse fast Fourier transform (IFFT) of the marked region are shown in Figure 4. The IFFT of the selected region (Figure 4b) revealed a periodic “zigzag” structure (highlighted by the red line), which is characteristic of crystallographic shear planes in reduced titanium oxide phases.^43,46^ This structure arises from the relaxation of Ti atoms in response to ordered oxygen vacancies and local rearrangements of [TiO_6_] corner-sharing to face-sharing octahedra motifs, leading to the formation of shear planes within the rutile lattice. The observed “zigzag” arrangement is thus a direct fingerprint of shear plane defects, in line with the structural analysis conducted with PXRD, Raman, and PDF. Additional images of Ti_0.50_Fe_0.50_O_1.75_ (Figure S12) and Ti_0.70_Fe_0.30_O_1.85_ (Figure S13) further support the presence of defects within the rutile structure. Additionally, HR-TEM of TiO_2_, Ti_0.90_Fe_0.10_O_1.95_, Ti_0.70_Fe_0.30_O_1.85_, and Ti_0.50_Fe_0.50_O_1.75_ (Figure S14) revealed that the morphology of the samples was highly correlated with their crystalline phase: anatase led to the formation of spheroidal nanoparticles, while rutile led to the formation of elongated rod-like shaped nanostructures.

**Figure 4:**
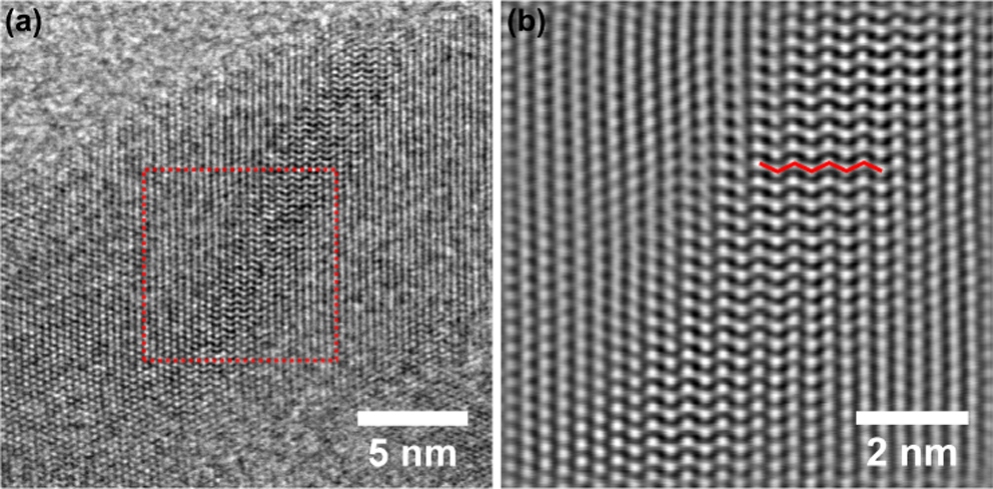
(a) Representative bright-field HR-STEM image of Ti_0.50_Fe_0.50_O_1.75_ and (b) IFFT of the highlighted region of (a). The red “zigzag” line in (b) is used to guide the eye for the defective region.

The phase fraction of anatase, rutile, and Ti_5_O_9_ (i.e., associated with shear planes) as a function of the Fe loading was extracted from the final PDF refinements (Figure 5a). Undoped TiO_2_ and Fe loadings up to 10 at. % Fe can be described predominantly as the anatase phase. At 15 at. % Fe, the anatase-to-rutile phase transition occurred, with anatase and rutile comprising 62% and 38%, respectively. The coexistence of anatase, rutile, and Ti_5_O_9_ was observed at 20 at. % Fe, with phase fractions of 12%, 66%, and 22%, respectively. Higher loadings of Fe led to only rutile and Ti_5_O_9_ phases, with Ti_5_O_9_ reaching a value of 74% at 50 at. % Fe, emphasizing the progressive formation of defects as the Fe concentration increased.

**Figure 5:**
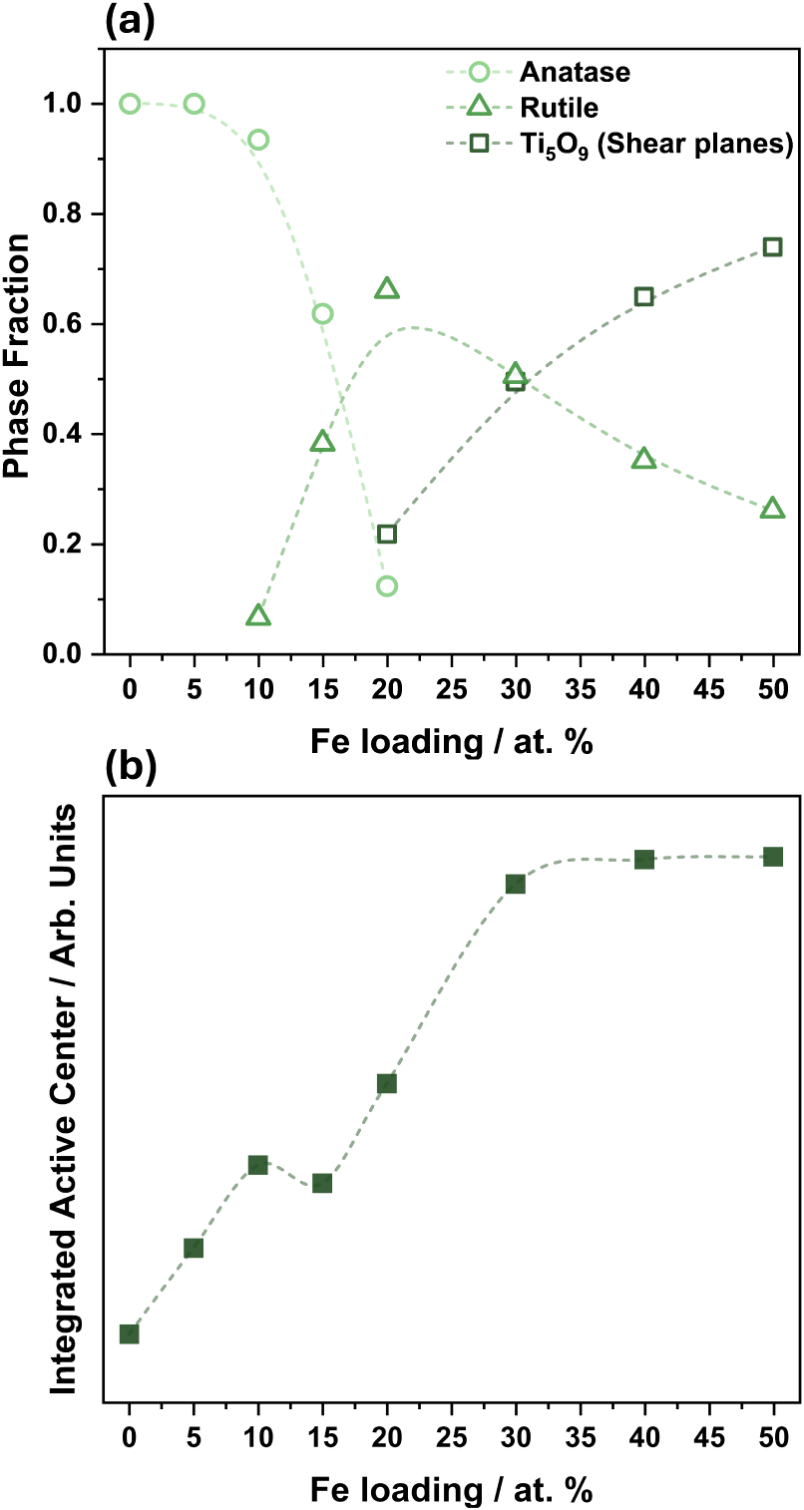
(a) Phase fraction extracted from PDF refinements as a function of the Fe loading, and (b) integrated EPR active center (i.e., associated with oxygen vacancies) as a function of the Fe loading. Data from (b) was obtained by integrating the EPR resonance at *g* = 2.0.

To further investigate the presence of oxygen defects in the samples, structural analysis was complemented by EPR measurements at room temperature. These revealed that the crystal structures of the samples led to different line shapes of the active center contribution (Figure S15a, *g* = 2.0), suggesting variations in its relaxation, stabilization, and/or conformation. EPR spectra of TiO_2_-based materials with active centers at *g* = 2.0 can be associated with paramagnetic centers of undercoordinated metal sites, O_2_ molecules adsorbed at oxygen vacancy sites as O_2_^–^, and/or trapped electrons on oxygen vacancy sites.^20,47–49^ Thus, the integrated area of the contribution at *g* = 2.0 was associated with the relative concentration of oxygen vacancies and is plotted as a function of the Fe loading in Figure 5b. Two distinct regimes of oxygen vacancy increase were observed along the Fe loading: the first one associated with the anatase phase and the second one with the rutile and Ti_5_O_9_ phases. In the first one, by increasing the loading of Fe in the anatase phase, a linear increase of oxygen vacancies occurred. The decrease in the oxygen vacancy concentration at 15 at. % Fe was associated with the anatase-to-rutile phase transition, corresponding to a Fe loading decrease in the anatase phase concomitantly with the incorporation of Fe in the rutile phase and/or leaching to the supernatant (Figures 1, S1, S2 and Table S1). The second regime was associated with the rutile phase containing defects (i.e., induced by shear planes), in which an increase in the oxygen vacancy concentration in samples up to 30 at. % Fe was observed. Higher loadings of Fe led to a plateau, demonstrating that a saturation limit of oxygen vacancies in the rutile structure was achieved.

To better understand the nature of the *g* = 2.0 signal and its evolution with Fe content, variable-temperature EPR measurements were conducted for selected samples (Figure S16) along with low-temperature spectra (5 K) for the full series (Figure S15b). At 5 K, the pure TiO_2_ sample exhibited a significantly saturated signal, presumably due to the extreme passage effects. This phenomenon occurs when field modulation is employed at a rate that is faster than the electron relaxation rate of the spin center.^50^ Paramagnetic relaxation enhancement, facilitated by the inclusion of rapidly relaxing iron centers, alleviated the saturation phenomenon observed in the signal.^51^ Additional Fe-related features below 2000 G only emerged for temperatures lower than 120 K for Ti_0.70_Fe_0.30_O_1.85_ (Figure S16b), indicating minimal contributions from isolated Fe centers at higher temperatures. While the possibility of superparamagnetic contributions in high Fe-loading samples (≥20 at. %) cannot be fully excluded,^52^ the lack of any deviation from a symmetrical line shape, along with the integrated area not following a linear increase with the Fe loading, suggests that such effects are not dominant.

To further support the formation of oxygen vacancies, XPS measurements of the O 1s core level were carried out for selected compositions (Ti_1–*x*_Fe_*x*_O_2–0.5*x*_, where *x* = 0, 0.1, 0.3, and 0.5). The O 1s spectra revealed two distinct species corresponding to lattice oxygen (O_L_, ca. 530 eV) and surface-adsorbed oxygen species (O_S_, ca. 531 eV), the latter commonly associated with surface oxygen vacancies.^53,54^ A progressive decrease in the O_L_/O_S_ ratio was observed with increasing Fe loading (Figure S17), consistent with the trend identified by EPR and indicating an increase in the surface oxygen vacancy concentration.

In summary, the analysis of the structural evolution of Fe-doped TiO_2_ nanostructures as a function of Fe loading showed that Fe induced a sequence of structural transformations from anatase at low Fe loadings to a defect-rich rutile phase at high concentrations. The generation of these defects is consistent with the increase in oxygen vacancy concentration and their collapse into a more thermodynamically stable conformation, which we associate with disordered shear planes. This process likely originates from local rutile domains enriched with ordered oxygen vacancies, which evolve into extended defects through shear plane formation.^12,55,56^ The increase in the defective phase upon Fe addition into the TiO_2_ lattice indicates that point defects are vital in the formation and dynamic propagation of extended defects induced by shear planes. This was elegantly demonstrated by Xue et al.,^13^ who probed the formation of Wadsley and Magnéli defects on TiO_2_ upon an external electrical field by *in situ* TEM. They revealed that the coalescence of point defects led to the formation of planar shear planes (i.e., Wadsley defects), and their stacking promoted the formation of the Magnéli phase. Understanding the structural evolution of these defects during synthesis is an important step toward controlling their formation. Thus, the impact of synthesis time was also explored here: first, targeting the formation of the defective phase (1–90 h), and second, at the earlier times (<1 h), where nucleation takes place.

### Defective Phase Evolution as a Function of Synthesis Time

The generation of extended defects is a process that occurs through multiple steps associated with migration, propagation, and ordering of point defects. Thus, to better understand the generation and structural evolution of the defects during the crystallization process, Ti_0.70_Fe_0.30_O_1.85_ was evaluated as a function of synthesis time, stopping the reaction after *t* = 1, 3, 10, 20, and 90 h (labeled as Ti_0.70_Fe_0.30_O_1.85_-*t*). The results and discussion in the previous section were based on samples obtained after 20 h of synthesis. ICP-OES measurements showed that about 70 at. % of the nominal amount of Fe was already incorporated in the solid phase at early synthesis times and did not evolve (Figure S18 and Table S6). PXRD data (Figure 6a) demonstrated that after 1 h, the sample was composed of nanocrystalline domains of anatase (ca. 2 nm, estimated by the Scherrer equation). The increase of the synthesis time to 3 h triggered the anatase-to-rutile phase transition, and the structure fully transformed to rutile at times ≥10 h. Raman analysis (Figure 6b) followed the same trend as the PXRD data; however, the anatase Raman modes evidenced narrowing and shifts to lower wavenumbers as a function of time (Table S7). This could be attributed to a decrease in the dispersion of the metal–oxygen bond distances and an increase in their overall value, consistent with the decrease in the amount of Fe in the anatase phase. Rutile Raman modes did not show significant variations in the Eg center and FWHM as a function of synthesis time (Table S7).

**Figure 6:**
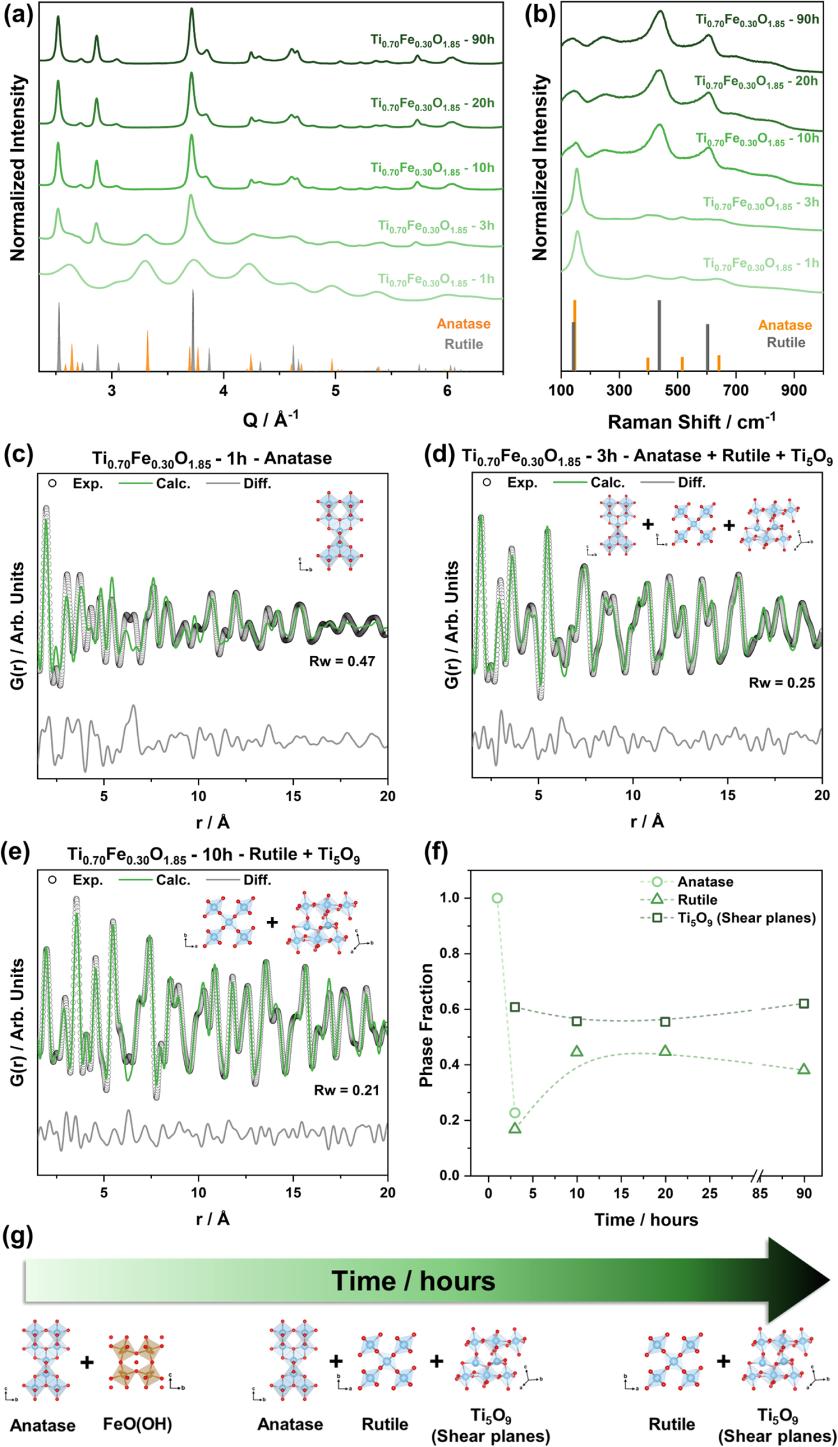
(a) Synchrotron PXRD and (b) Raman data of Ti_0.70_Fe_0.30_O_1.85_ nanostructures as a function of synthesis time; short- to medium-range PDF refinements of (c) Ti_0.70_Fe_0.30_O_1.85_-1h, (d) Ti_0.70_Fe_0.30_O_1.85_-3h, and (e) Ti_0.70_Fe_0.30_O_1.85_-10h; (f) phase fraction extracted from PDF refinements as a function of the synthesis time. The phases employed for each refinement are shown at the top of the plots. (g) Scheme of the structural evolution as a function of synthesis time.

PDF analysis of the short- to medium-range was also employed in this set of samples. Anatase was used as the model for Ti_0.70_Fe_0.30_O_1.85_-1h (Figure 6c); however, the goodness of the fit and the residual PDF indicate that the structure could not be fully described. Additional analysis of the residual PDF suggests that the sample also contained a mixture of amorphous domains of Fe (oxy)­hydroxides (Figure S19). A multiphase refinement using anatase, rutile, and Ti_5_O_9_ as models described Ti_0.70_Fe_0.30_O_1.85_-3h (Figure 6d), and a combination of rutile and Ti_5_O_9_ was enough to describe Ti_0.70_Fe_0.30_O_1.85_-10h (Figure 6e) and longer synthesis times (Figure S20). The refined parameters are presented in Table S8. The phase fraction as a function of the synthesis time (Figure 6f) depicts the structural evolution of Ti_0.70_Fe_0.30_O_1.85_. The first hour of synthesis resulted in the crystallization of anatase nanodomains alongside a mixture of amorphous Fe (oxy)­hydroxides. As the synthesis progressed, the anatase-to-rutile phase transition occurred, with the concomitant formation of defects induced by shear planes. At longer times, there was a complete formation of rutile containing defects, with no significant variation in the density of defects as a function of synthesis time. The scheme in Figure 6g summarizes these findings.

The synthesis time evaluation of Ti_0.70_Fe_0.30_O_1.85_ (Figure 6) sheds light on structural transformations for stabilizing oxygen vacancies. It indicates that the initial nanosized anatase crystalline domains only partially incorporated Fe, as anatase coexisted with amorphous Fe (oxy)­hydroxides. The depletion of Fe in the anatase phase and enrichment in the rutile and/or defective phase as a function of synthesis time suggest that the phase transformations were triggered by a critical concentration of Fe in the anatase phase. As the anatase particle size increases, more Fe is incorporated until it reaches this threshold, inducing the anatase-to-rutile phase transition and concurrent formation of defects. This is consistent with the consumption of the Fe (oxy)­hydroxides and their incorporation into the rutile and defective phases. As the incorporation of Fe in the TiO_2_ lattice is directly correlated with the formation of oxygen vacancies, this hypothesis can be corroborated by the elegant work reported by Drozd et al.^12^ They proposed a three-step structural evolution for anatase nanoparticles containing oxygen vacancies: (1) generation of oxygen vacancies with Ti-surrounding cation repulsion; (2) movement and ordering of oxygen vacancies to a more stable position; and (3) collapse of oxygen vacancies and formation of a defective phase. Thus, we assume that the formation of the rutile phase and extended defects occurs simultaneously.

### Nucleation and Growth of Fe-Doped TiO_2_ Nanomaterials

*In situ* X-ray TS and PDF analysis under hydrothermal synthesis conditions were employed to investigate the material formation and crystallization processes at early synthesis time (<1 h), providing insights into the nucleation and growth mechanisms of TiO_2_ nanoparticles in the presence of Fe. Figure 7 displays an overview of the in-depth analysis of two selected samples, TiO_2_ and Ti_0.70_Fe_0.30_O_1.85_. The synthesis media of TiO_2_ was composed of a basic solution of titanium lactate, while for Ti_0.70_Fe_0.30_O_1.85_, an iron oxalate complex was added to the mixture. The time-resolved PDF contour plot of TiO_2_ is presented in Figure 7a. The initial PDF showed medium-range order features, with correlations up to ca. 20 Å, assigned to the synthesis structural precursor. A fast crystallization process took place during heating (Figure 7c), leading to the formation of anatase-phase nanoparticles with mean crystallites of ca. 65 Å after 20 min (300 °C, Figure S21). For Ti_0.70_Fe_0.30_O_1.85_ (Figure 7b), on the other hand, a region associated with crystalline domains of a mixture of iron (oxy)­hydroxides (Figure S22) could also be observed at the initial stages, consistent with the data presented in Figures 6c and S19. Upon heating (after ca. 3 min, 70 °C), these iron-containing species disappeared, and the synthesis followed a pathway similar to that observed for TiO_2_. PDF refinements of *t* = 5 min (TiO_2_ structural precursor and Ti_0.70_Fe_0.30_O_1.85_ intermediate) and *t* = 40 min (crystalline nanoparticles) are presented in Figure 7d,e. The analysis revealed that the synthesis intermediate of Ti_0.70_Fe_0.30_O_1.85_ (i.e., after the dissolution of the iron (oxy)­hydroxides) exhibits features that resemble the anatase phase, suggesting the formation of a TiFeO_*x*_ intermediate with an anatase-like structure. After crystallization, the fit quality increased, and the PDFs of both materials were well-described by the anatase model. Pearson correlation of the experimental data (Figure 7f,g) provides a visual way of looking at the data qualitatively and extracting information regarding the different synthesis events. The red regions of the plot represent highly correlated PDFs (i.e., close to 1.0), while the blue ones represent less correlated PDF data. For pure TiO_2_, it was possible to observe two highly correlated regions: the first related to the precursors in solution (up to ca. 10 min, 200 °C) and the second related to the crystalline TiO_2_ anatase particles (ca. 15–40 min, 280–300 °C + 300 °C isotherm). The “bottleneck” connection between the two regions (ca. 10–15 min, 200–280 °C) is associated with the crystallization and growth process. The addition of Fe led to a wider, highly correlated region, located in the “bottleneck” (ca. 13–17 min, 250–300 °C, Figure 7g). We attribute this to an intermediate phase formed in the presence of Fe, as highlighted by the differential PDF data from Ti_0.70_Fe_0.30_O_1.85_ – TiO_2_ (Figure S23). Additionally, the differential PDF also revealed that the iron (oxy)­hydroxides at the beginning of the synthesis dissolved into a structure similar to the iron oxalate precursor after ca. 3 min of synthesis time (compare black and blue PDFs of Figure S23).

**Figure 7:**
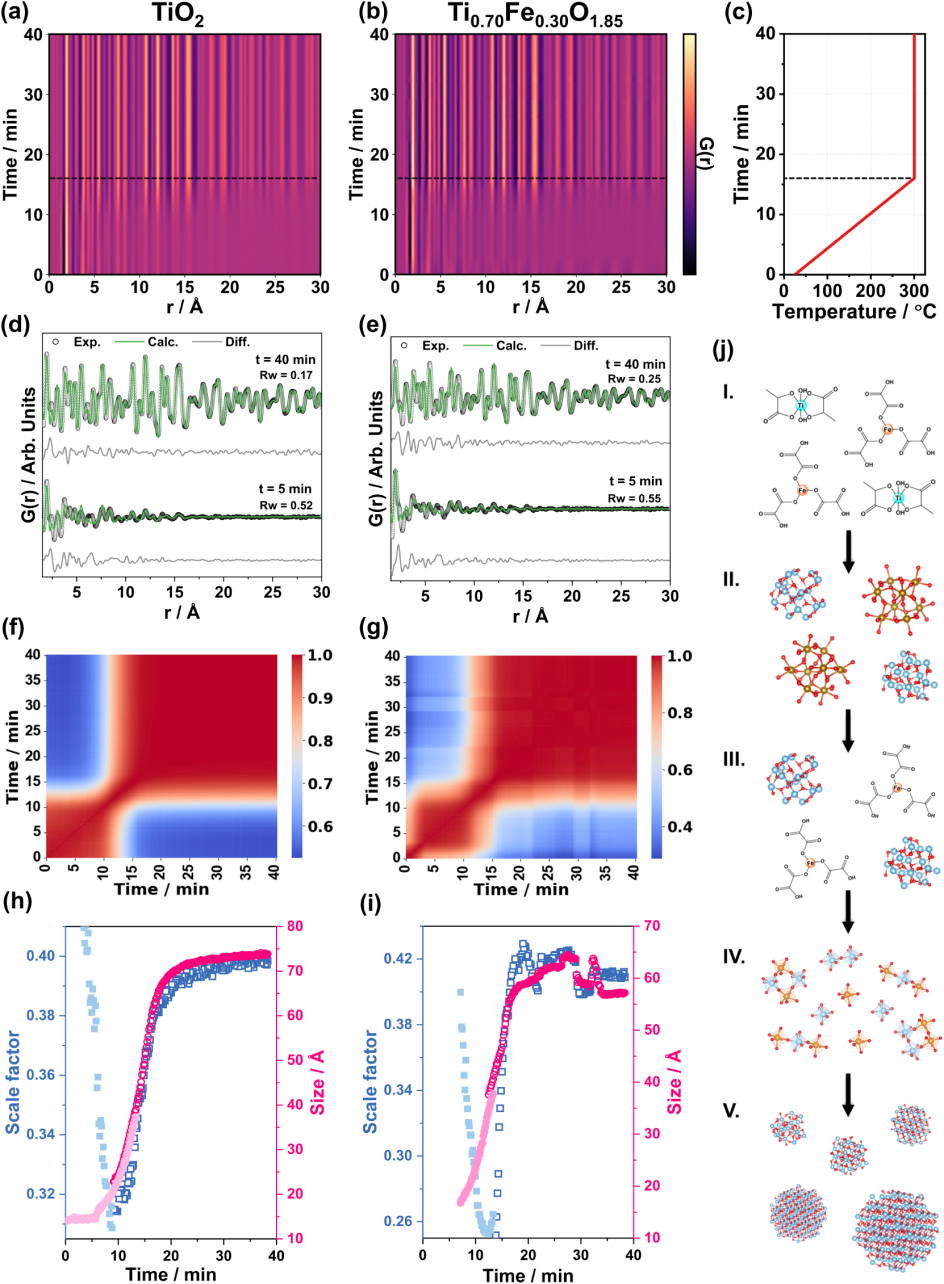
PDF analysis of time-resolved *in situ* hydrothermal data of TiO_2_ and Ti_0.70_Fe_0.30_O_1.85_ comprising (a,b) experimental PDF contour plots, (c) heating profile of the (a,b) contour plots, (d,e) short- to medium-range PDF refinements of t = 5 min (synthesis precursor) and t = 40 min (final crystalline material) stages using the TiO_2_ anatase crystal structure as model, (f,g) Pearson correlation plots of experimental PDFs, (h,i) refined scale factors (crystallization) and mean crystallite sizes (growth) as a function of reaction time, and (j) scheme of the proposed synthesis mechanism of Ti_0.70_Fe_0.30_O_1.85_. In (h,i), filled symbols are associated with water background discount data, whereas open symbols correspond to a combination of water and air.

The particle formation mechanism can be assessed by the evolution of two PDF fitting parameters: the scale factor and crystallite size.^57^ The scale factor is proportional to the number of scatterers that the structure model describes (i.e., the number of nuclei or crystallites). On the other hand, the size is related to the coherent crystalline domain size (i.e., crystallite size). Sequential PDF refinements provided time-resolved scale factor (crystallization) and size (growth) parameters (Figure 7h,i), allowing tracking of the nucleation and growth mechanisms of the particles. The refined scale parameter showed that both samples were initially composed of precursor nuclei that partially dissolved upon heating (i.e., a decrease in the scale factor) and crystallized into the final anatase particles in less than 20 min. The integrated area of the corner-sharing octahedra’s metal–metal atomic pair (ca. 3.7 Å) as a function of time qualitatively corroborates this phenomenon (Figure S24). During the crystallization process (ca. 10–15 min, 200–280 °C) of pure TiO_2_, the scale factor and the crystallite size rise simultaneously (Figure 7h), which suggests that the particles grow due to crystallization in a conventional nucleation and growth process. The addition of 30 at. % Fe led to similar profiles (Figure 7i); the small oscillations of the data are likely attributed to experimental artifacts. A schematic representation of the proposed synthesis mechanisms of Ti_0.70_Fe_0.30_O_1.85_ is presented in Figure 7j, and its comparison with the Pearson correlation (Figure 7g) is depicted in Figure S25.

*In situ* hydrothermal PDF data provided insights into the synthesis mechanisms and the insertion of Fe into the TiO_2_ lattice. From the overall analysis, we can now describe the steps of the reaction, as illustrated in Figure 7j: (I) first, metallic complexes existed in solution as titanium lactate and iron oxalate; (II) the basic medium of (I) led to the formation of crystalline Fe hydroxides and a TiFeO_*x*_ intermediate composed of a disordered phase with anatase-like motifs; (III) heating of (II) induced dissolution of the Fe (oxy)­hydroxides at ca. 70 °C; and (IV) further heating led to the partial dissolution of the TiFeO_*x*_ intermediates. This is followed by the formation of another intermediate comprising [TiO_6_] and/or [FeO_6_] motifs in the 140 to 250 °C range, which was followed by a quick crystallization into the Fe-doped TiO_2_ nanoparticles of (V).

## Conclusions

In this work, we provide a comprehensive investigation of the structural evolution of Fe-doped titanium oxide nanomaterials, emphasizing the role of oxygen vacancies in driving phase transformations and defect formation. Focusing on a moderate Fe concentration (30 at. %), we show that, at the early stages of synthesis, Fe (oxy)­hydroxide species and disordered TiFeO_*x*_ intermediates form prior to crystallization. These intermediates gradually dissolve, preceding the nucleation of the anatase nanoparticles and the progressive incorporation of Fe into the TiO_2_ lattice. As synthesis progresses, Fe is gradually incorporated into the anatase lattice until a critical threshold is reached, triggering the anatase-to-rutile phase transition. At longer synthesis times, the structure transforms into rutile, and the high concentration of oxygen vacancies collapses into disordered shear planes, which stabilize the oxygen-deficient lattice.

A systematic evaluation of the Fe loading reinforces this structural evolution. At low concentrations (≤10 at. %), Fe is incorporated into the anatase lattice, while increasing the content to intermediate concentrations (15–20 at. %) promotes the anatase-to-rutile phase transition. At higher concentrations (≥30 at. %), the structure evolves into rutile containing a high density of defects associated with disordered shear planes. These extended defects play a central role in accommodating high Fe concentrations and facilitate the incorporation of dopants beyond the conventional limits of solid solution formation.

In summary, we have elucidated the structural evolution of Fe-doped titanium oxide nanomaterials, shedding light on their synthesis, phase transformations, and defect formation. These findings demonstrate that structural evolution dynamics in the nano regime are dictated by the interplay between dopant incorporation, oxygen vacancy distribution, and defect formation. Disordered shear planes emerge as a structural response to oxygen deficiency, stabilizing nonstoichiometric lattices and incorporating dopant concentrations beyond those typically achieved for solid solutions. These insights provide a generalized understanding of how defect formation facilitates dopant incorporation at the nanoscale and offer strategies for defect engineering across a broader class of reducible oxide nanomaterials.

## Methods

### Chemicals

Titanium­(IV) bis­(ammonium lactate) dihydroxide 50 wt % in H_2_O (TALH), ammonium iron­(III) oxalate (AIO), ammonium hydroxide 28–30% (NH_4_OH), and titanium and iron ICP standards (1000 mg L^–1^) were purchased from Sigma-Aldrich. Ethanol 96% and nitric acid 69% (AnalaR NORMAPUR analytical reagent, HNO_3_) were purchased from VWR Chemicals.

### Synthesis of Fe-Doped TiO_2_ Nanomaterials

The hydrothermal synthesis of Ti_1–*x*_Fe_*x*_O_2–0.5*x*_ nanostructures with various Ti:Fe at. ratios (final metal concentration of 0.2 M) consisted of: (1) the dissolution of the desired amount of AIO in 20 mL of H_2_O, resulting in a lime-green limpid solution and a slight decrease in the pH; (2) the addition of the desired volume of TALH under stirring, resulting in no apparent color change and a slight increase in the pH; (3) the addition of NH_4_OH dropwise up to a concentration of 0.15 M (final volume of 22.5 mL), under vigorous stirring, resulting in color changes from yellow to orange to brownish with the Fe loading increase. The final pH varied from 9.3 to 8.6 with increasing Fe loading. For instance, for Ti_0.85_Fe_0.15_O_1.92_, 266.4 mg of AIO was dissolved in 20 mL of H_2_O (pH = 5.2), followed by the addition of 1.7 mL of TALH under stirring (pH = 5.6), and the addition of 0.4 mL of NH_4_OH dropwise under vigorous stirring (pH = 9.1). The solution was then placed in a 45 mL Teflon-lined steel autoclave and heated at 160 °C for 20 h. The obtained solid was centrifuged at 9000 rpm for 5 min, the supernatant was removed, and the solid was redispersed in distilled water. It was dried at 100 °C overnight, followed by grinding and calcination at 400 °C for 1 h. The same procedure was used to evaluate the impact of the synthesis time.

### Powder X-ray Diffraction and X-ray Total Scattering

Powder X-ray diffraction (PXRD) and X-ray total scattering (TS) measurements were performed at the P02.1 PETRA III beamline at the DESY (Hamburg, Germany), using a wavelength of λ = 0.20734 Å, and at the DanMax beamline at MaxIV (Lund, Sweden), using a λ = 0.35424 Å. The powder was packed in 1 mm diameter Kapton capillaries for PXRD and TS data collection. The Rapid Acquisition Pair Distribution Function (RA-PDF) setup was used for all TS experiments^58^ The 2D data were integrated using PyFAI,^59^ and the TS data were Fourier transformed to obtain PDFs using PDFgetX3.^60^ A Q-range from 1.2 to 22 Å^–1^ and an *r*_poly_ of 0.9 were employed for the *ex situ* measurements from the P02.1 beamline, while a Q-range from 1.0 to 15.5 Å^–1^ and an *r*_poly_ of 1.2 were used for the *ex situ* and *in situ* measurements from the DanMax beamline. *In situ* hydrothermal X-ray TS experiments were carried out in a custom-made reaction cell, similar to the design described by Becker et al.,^61^ at the DanMax beamline. TALH and AlO precursors were dissolved in H_2_O (total metal concentration of 1 M), followed by the addition of NH_4_OH (final concentration of 0.75 M), and injected into a fused silica capillary with a 0.7 mm inner diameter and 0.09 mm wall thickness. It was pressurized to 2000 psi using an HPLC pump and heated to 300 °C (17 °C min^–1^) using a hot air blower. Before the Fourier transformation, the background scattering signal from the fused silica capillary, air, and the pure solvent at the appropriate temperature and pressure was subtracted. The PDFs were analyzed with real-space Rietveld refinements using PDFgui^62^ for both *ex situ* and *in situ* measurements. The experimental section of the Supporting Information discusses the refinement strategies in more detail. Le Bail refinements were employed to estimate the mean crystallite size and lattice parameters from the PXRD data using GSAS-II.^63^

### Raman Scattering

Raman spectra were acquired using an inVia confocal Raman microscope and the Wire v. 5.4 software (Renishaw, Gloucestershire, UK) with a CCD detector. The powder samples were measured over a microscope glass slide with a laser excitation of 785 nm, a laser power of 10 mW, dispersed by a 1200 lines mm^–1^ grating, and an exposure of 10 s over 3 accumulations. Data were collected with a spectral range from 100 to 1000 cm^–1^ with a spectral resolution of 1 cm^–1^.

### High-Resolution (Scanning) Transmission Electron Microscopy

HR-(S)­TEM images were collected on a Tecnai T20 or a Titan Cubed Themis microscope. A single-tilt sample holder was used for sample collection in both pieces of equipment. Aberration-corrected STEM images were collected on an FEI Titan Cubed Themis microscope. The samples were suspended by sonication in ethanol. Ten μL of the suspension was drop-casted on Quantifoil Cu 300 TEM grids and air-dried. The images were acquired using an acceleration voltage of 200 kV on a Tecnai T20 and 300 kV on a Titan Cubed Themis.

### Electronic Paramagnetic Resonance

EPR measurements were performed at the Novo Nordisk Foundation Copenhagen Pulse EPR Facility using a Bruker Elexsys E580 spectrometer (X-band, ∼9.6 GHz) equipped with a Flexline MD-5 dielectric resonator. Samples were placed in 4 mm outer diameter (O.D.) quartz tubes and were recorded open to the air at room temperature or at low temperature (i.e., 5 K) using liquid He. Temperature-dependent measurements were also conducted, from room temperature to 10 K. Spectra were recorded using 1.5 mW microwave power, 8192 field points, an 84 s field sweep time, and 1 G field modulation at a rate of 100 kHz, with a lock-in integration conversion time of 10.24 ms. Spectra shown were an average of 5 field sweeps. The double integral intensity was obtained via sequential cumulative trapezoidal numerical integration in MATLAB (MathWorks). After the first integration, a linear baseline correction was performed by subtraction of a linear fit to the baseline using an asymmetric truncated quadratic function (1% threshold) in MATLAB (“backcor”).^64^ The double integral intensities were scaled accordingly to account for slight differences in the sample height.

### X-ray Photoelectron Spectroscopy

XPS measurements were carried out using a monochromatic Al Kα source (Kα = 1486 eV) on a K-alpha spectrometer (Thermo Fisher Scientific) equipped with a 180° double-focusing hemispherical analyzer with a 128-channel detector. Selected high-resolution atomic spectra were obtained with a pass energy of 50 eV, an energy step of 0.1 eV, and a spot size of 300 μm. The Casa XPS software package was used to analyze the spectra, and the peaks were fitted using a mixed Gauss–Lorentz function and Shirley background. All of the measured spectra were corrected by setting the reference binding energy of carbon (C 1s) at 285 eV.

### Inductively Coupled Plasma Optical Emission Spectroscopy

The inductively coupled plasma optical emission spectroscopy (ICP-OES) measurements were carried out on an iCAP PRO ICP-OES instrument with an automated stage from Thermo Fisher Scientific. The emission wavelengths employed for the measurements were chosen to avoid overlap from Ti and Fe. Three measurements of each sample were averaged. The resultant concentrations were obtained from a combination of all of the measured emission lines. A calibration curve employing Ti and Fe standards was obtained to calculate the concentration of the samples, carried out on Qtegra software. The supernatant of the syntheses was diluted in a solution of HNO_3_ 10% v/v before performing the measurements. The actual concentration of the metals (i.e., Ti and Fe) in the synthesized samples was calculated by subtracting the residual metals in the supernatant from the known amount employed in each synthesis.

## Supplementary Material



## Notes

Complementary detailed information about the materials characterization and data analysis, and additional characterization comprising ICP-OES, HR-TEM, PXRD, PDF, and XPS (PDF)
