# Supplementary material for: Oxygen Vacancy-Induced Phase Transformations of Iron-Doped Titanium Oxide Nanostructures
Source: ACS Nano. 2025 Aug 21;19(34):30986–99. doi: 10.1021/acsnano.5c08093 (PMC12410056; doi:10.1021/acsnano.5c08093)
Supplement: Supplementary file 1 [file nn5c08093_si_001.pdf]

Supporting Information

Oxygen vacancy-induced phase transformations of iron-doped titanium oxide nanostructures

Guilherme B. Strapasson[1,2,3], Adrián S. Arjona[3], Joseph E. McPeak[3], Olivia Aalling-Frederiksen[3], Adam F. Sapnik[3], Nanna L. Baun[3], Heloisa N. Bordallo[4], Cristiane B. Rodella[2,*], Daniela Zanchet[1,*], and Kirsten M. Ø. Jensen[3,*]

[1] Institute of Chemistry, Universidade Estadual de Campinas, UNICAMP, Campinas, SP 13083-970, Brazil

[2] Brazilian Synchrotron Light Laboratory, CNPEM, Campinas, SP 13083-100, Brazil

[3] Department of Chemistry, University of Copenhagen, Universitetsparken 5, 2100 Copenhagen Ø, Denmark

[4] Niels Bohr Institute, University of Copenhagen, Universitetsparken 5, 2100 Copenhagen Ø, Denmark

* Corresponding Authors: kirsten@chem.ku.dk; zanchet@unicamp.br; cristiane.rodella@lnls.br

EXPERIMENTAL SECTION

Real-space Rietveld refinement strategies. The refined parameters included the scale factor, lattice parameters, spherical particle diameter, correlated atomic motion, and isotropic atomic displacement parameters (ADPs) for each metal present. Q_{damp} and Q_{broad} values were obtained by refining Si and LaB_6 NIST standards. The refinements were carried out by employing single or multiple-phase models with anatase TiO_2 (ICSD collCode 56447), rutile TiO_2 (ICSD collCode 121630), Ti_3O_5 (ICSD collCode 35148), Ti_5O_9 (ICSD collCode 9038), and $TiFe_2O_5$ (ICSD collCode 24416). The metal occupancy of the models (*i.e.*, Ti or Fe) was modified according to each nominal sample composition.

Background subtraction of the *in situ* TS data was divided into two sections: **(1)** water background discount before the crystallization of the nanoparticles at the appropriate temperature and pressure; **(2)** a linear combination of air and water at the appropriate temperature and pressure during and after the nanoparticle's crystallization process. This approach was necessary due to bubble generation during crystallization at temperatures ≥ 250 °C, thus accounting for the air and water inside the capillary. Direct sequential refinements were applied to **(1)**, whereas inverse sequential refinements were employed for **(2)**.

Pearson Correlation Analysis. To facilitate data analysis and better understand correlations and differences between datasets, we introduce straightforward tools based on Pearson Correlation Coefficient (PCC) analysis [1]. The PCC quantifies the degree of linear correlation between two datasets and, in this context, reflects the similarity between two PDFs. It ranges from -1 to 1, where a value of 1 indicates perfect linear correlation, and -1 describes the complete opposite behavior between the two functions. This measure is scale-invariant, meaning it accounts only for peak positions and widths, making it a fast and straightforward method for comparing PDFs or other datasets. We apply the PCC to the measured PDFs (i.e., the correlation between the PDFs with each other) to gain an overall view of the time-resolved *in situ* data and to identify structural changes occurring during the synthesis process. The same approach was employed for comparing experimental PDFs with simulated PDFs of crystal structures from the Inorganic Crystal Structure Database (ICSD) database.

RESULTS AND DISCUSSION

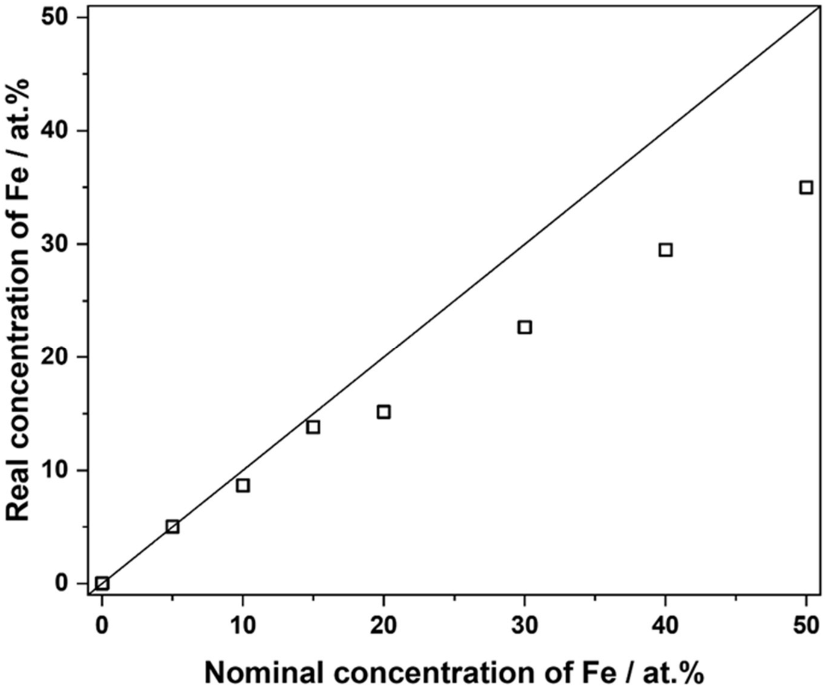

Figure S1: Correlation of the nominal (black line) and real concentrations (open squares) of Fe (at. %) of the $Ti_{1-x}Fe_xO_{2-0.5x}$ samples as a function of Fe concentration (*i.e.,* $0 \leq x \leq 0.5$).

Table S1: Nominal and real concentrations of Fe (at.%) of the $Ti_{1-x}Fe_xO_{2-0.5x}$ samples as a function of Fe concentration (*i.e.,* $0 \leq x \leq 0.5$).

Sample	Nominal concentration of Fe / at.%	Real concentration of Fe / at.%
TiO_2	0	0
$Ti_{0.95}Fe_{0.05}O_{1.97}$	5	5
$Ti_{0.90}Fe_{0.10}O_{1.95}$	10	8.7
$Ti_{0.85}Fe_{0.15}O_{1.92}$	15	13.8
$Ti_{0.80}Fe_{0.20}O_{1.90}$	20	15.2
$Ti_{0.70}Fe_{0.30}O_{1.85}$	30	22.7
$Ti_{0.60}Fe_{0.40}O_{1.80}$	40	29.5
$Ti_{0.50}Fe_{0.50}O_{1.75}$	50	35

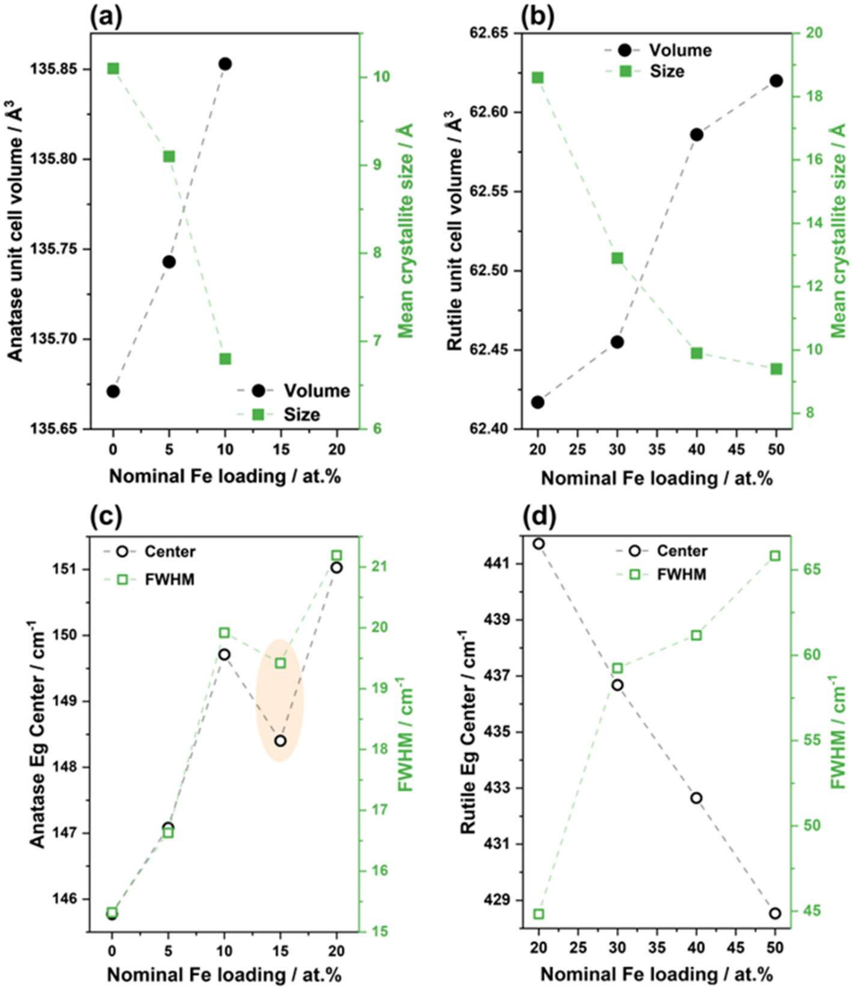

Figure S2: Correlations of the (a) anatase unit cell volume and mean crystallite size, (b) rutile unit cell volume and mean crystallite size, (c) anatase Eg center and respective FWHM, and (d) rutile Eg center and respective FWHM as a function of the nominal Fe loading (at. %).

Table S2: Structural parameters obtained by Le Bail refinements using anatase as model.

Sample	Rwp (%)	a-lattice (Å)	c-lattice (Å)	Volume ($Å^3$)	Mean crystallite size (nm)
TiO_2	6.1	3.782	9.486	135.67	10.1
$Ti_{0.95}Fe_{0.05}O_{1.97}$	4.5	3.784	9.481	135.74	9.1
$Ti_{0.90}Fe_{0.10}O_{1.95}$	4.7	3.786	9.478	135.85	6.8

Table S3: Structural parameters obtained by Le Bail refinements using rutile as model.

Sample	Rwp (%)	a-lattice (Å)	c-lattice (Å)	Volume ($Å^3$)	Mean crystallite size (nm)
$Ti_{0.80}Fe_{0.20}O_{1.90}$	11.7	4.598	2.952	62.417	18.6
$Ti_{0.70}Fe_{0.30}O_{1.85}$	11.6	4.602	2.949	62.455	12.9
$Ti_{0.60}Fe_{0.40}O_{1.80}$	12.0	4.610	2.945	62.586	9.9
$Ti_{0.50}Fe_{0.50}O_{1.75}$	11.6	4.610	2.946	62.62	9.4

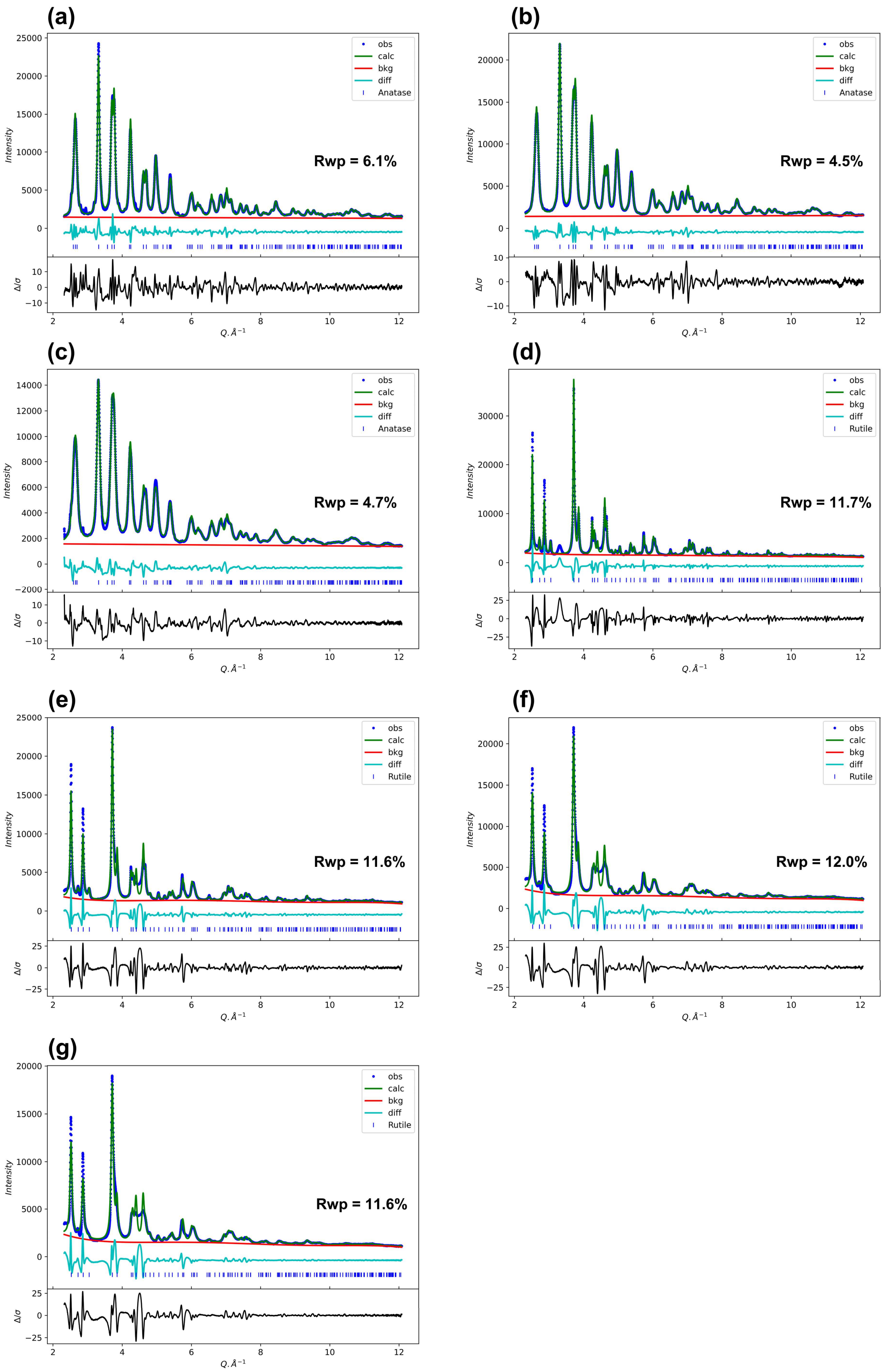

Figure S3: Le Bail refinements of (a) TiO_2, (b) $Ti_{0.95}Fe_{0.05}O_{1.97}$, (c) $Ti_{0.90}Fe_{0.10}O_{1.95}$, (d) $Ti_{0.80}Fe_{0.20}O_{1.90}$, (e) $Ti_{0.70}Fe_{0.30}O_{1.85}$, (f) $Ti_{0.60}Fe_{0.40}O_{1.80}$, and (g) $Ti_{0.50}Fe_{0.50}O_{1.75}$ using anatase or rutile.

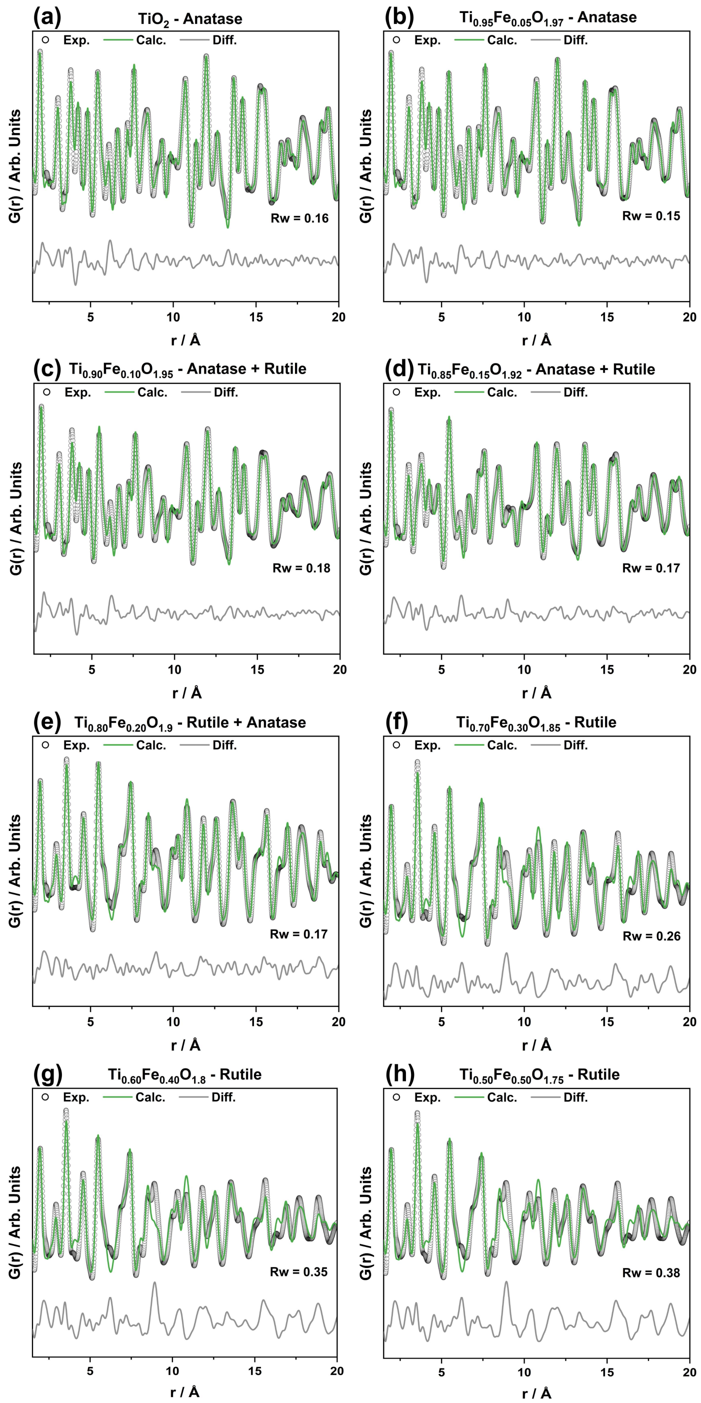

Figure S4: Short- to medium-range PDF refinements from *ex situ* measurements of (a) TiO_2, (b) $Ti_{0.95}Fe_{0.05}O_{1.97}$, (c) $Ti_{0.90}Fe_{0.10}O_{1.95}$, (d) $Ti_{0.85}Fe_{0.15}O_{1.92}$, (e) $Ti_{0.80}Fe_{0.20}O_{1.90}$, (f) $Ti_{0.70}Fe_{0.30}O_{1.85}$, (g) $Ti_{0.60}Fe_{0.40}O_{1.80}$, (h) $Ti_{0.50}Fe_{0.50}O_{1.75}$. Anatase and rutile TiO_2 polymorphs were used as models for the refinements. The phases employed for each refinement are shown at the top of the plots.

Table S4: PDF real space Rietveld refinement parameters of $Ti_{1-x}Fe_xO_{2-0.5x}$ (*i.e.*, $0 \leq x \leq 0.5$) employing anatase, rutile, or a combination of anatase + rutile as model.

Sample	Rw	Phase model	Scale factor	δ2 (Å^2)	a=b lattice parameter (Å)	c lattice parameter (Å)	ADP_{Ti} (Å^2)	ADP_{Fe} (Å^2)	ADP_{O} (Å^2)
TiO_2	0.16	Anatase	0.58	2.06	3.7834	9.4864	0.0060	-	0.0153
$Ti_{0.95}Fe_{0.05}O_{1.97}$	0.15	Anatase	0.60	2.03	3.7873	9.4980	0.0059	0.0070	0.0156
$Ti_{0.90}Fe_{0.10}O_{1.95}$	0.18	Anatase	0.57	2.22	3.7869	9.4895	0.0066	0.0056	0.0164
		Rutile	0.04	2.00	4.5452	2.9941	0.0052	0.0196	0.0807
$Ti_{0.85}Fe_{0.15}O_{1.92}$	0.17	Anatase	0.39	2.17	3.7875	9.4876	0.0061	0.0060	0.0165
		Rutile	0.24	2.76	4.5892	2.9620	0.0082	0.0083	0.0470
$Ti_{0.80}Fe_{0.20}O_{1.90}$	0.17	Anatase	0.07	1.73	3.7916	9.4765	0.0047	0.0053	0.0252
		Rutile	0.55	2.29	4.5963	2.9578	0.0073	0.0081	0.0216
$Ti_{0.70}Fe_{0.30}O_{1.85}$	0.26	Rutile	0.67	2.24	4.5961	2.9576	0.0081	0.0080	0.0240
$Ti_{0.60}Fe_{0.40}O_{1.80}$	0.35	Rutile	0.68	2.43	4.5970	2.9579	0.0087	0.0096	0.0291
$Ti_{0.50}Fe_{0.50}O_{1.75}$	0.38	Rutile	0.69	2.46	4.5968	2.9584	0.0087	0.0103	0.0300

* Short- to medium-range order real space Rietveld refinements primarily reflect local structural order rather than the full nanoparticle size, thus mean particle size diameter was not included in the refined parameters table.

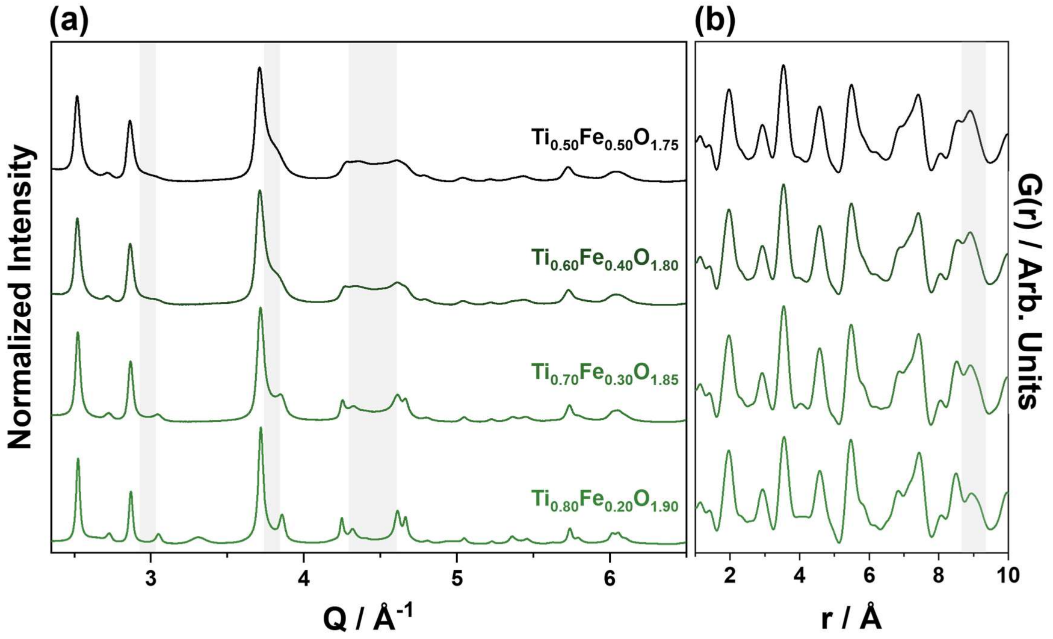

Figure S5: Synchrotron PXRD and PDF data of $Ti_{1-x}Fe_xO_{2-0.5x}$ nanostructures with Fe loadings from $x = 0.20$ to $x = 0.50$. Gray areas highlight defective phase region fingerprints.

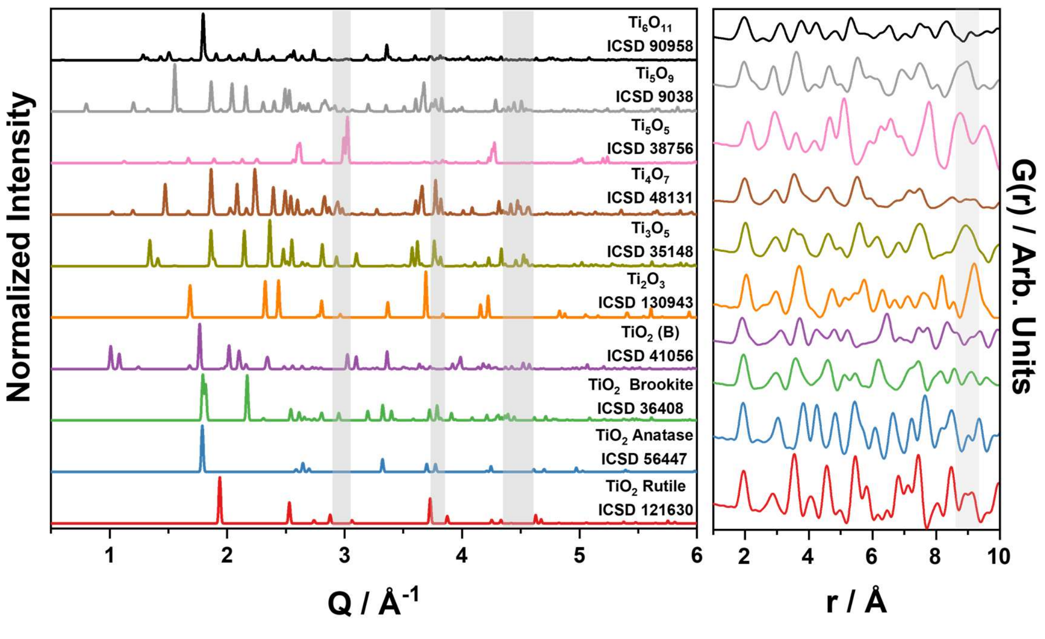

Figure S6: Simulated XRD and PDF data from multiple titanium oxide phases. Gray areas highlight defective phase region fingerprints.

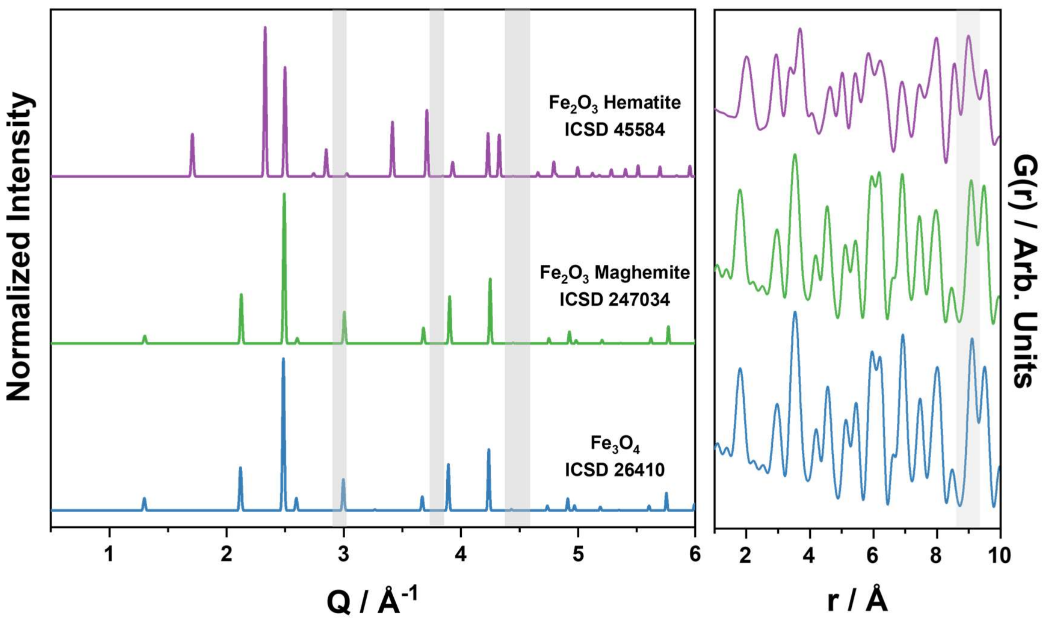

Figure S7: Simulated XRD and PDF data from multiple iron oxide phases. Gray areas highlight defective phase region fingerprints.

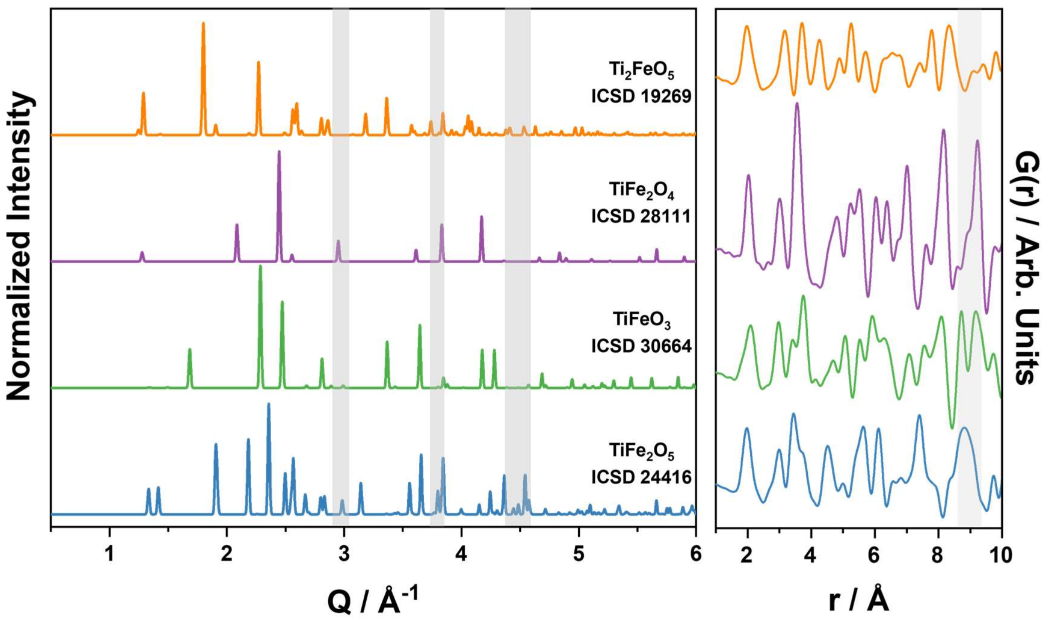

Figure S8: Simulated XRD and PDF data from multiple titanium-iron oxide phases. Gray areas highlight defective phase region fingerprints.

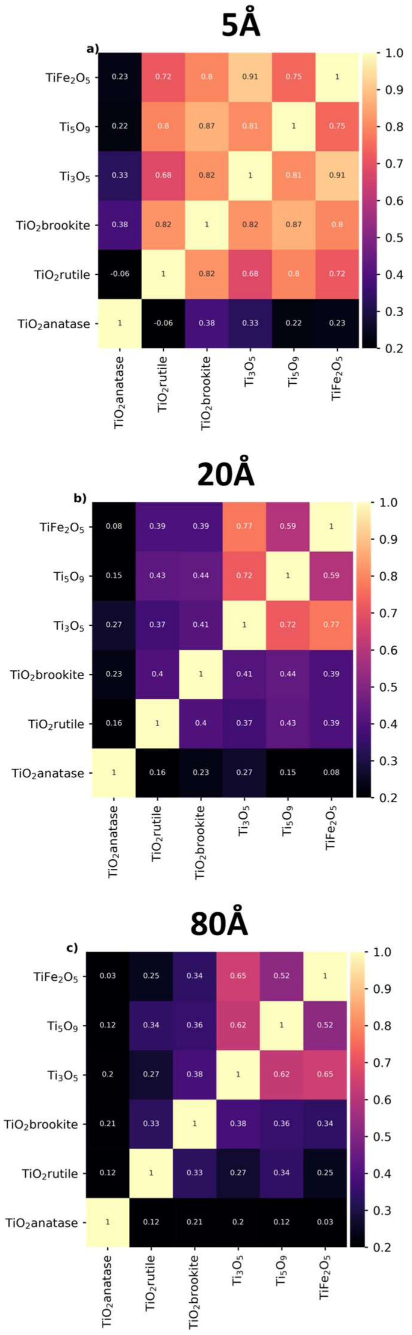

Figure S9: Pearson correlation analysis of simulated PDFs from TiO_2 polymorphs (anatase, rutile, and brookite), Ti_3O_5, Ti_5O_9, and $TiFe_2O_5$ employing *r* ranges of (a) 5 Å, (b) 20 Å, and (c) 80 Å.

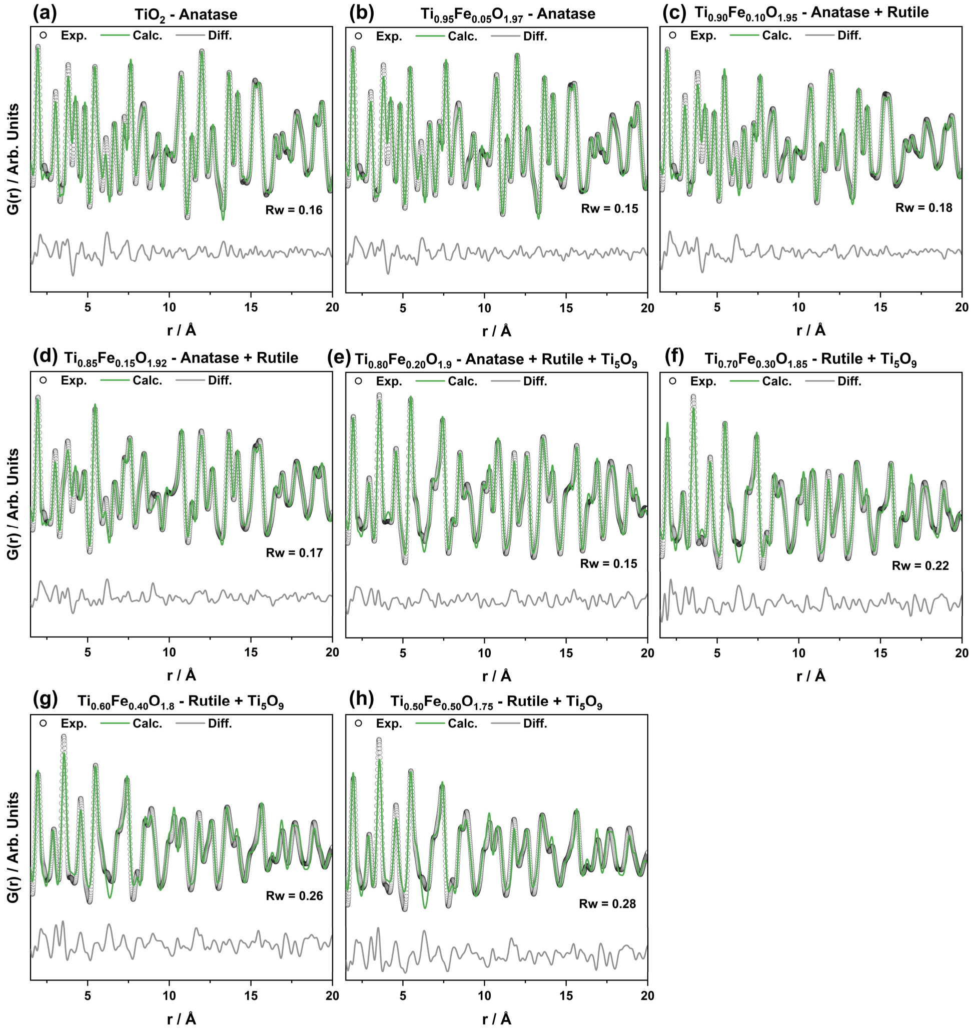

Figure S10: Short- to medium-range PDF refinements from *ex situ* measurements of (a) TiO_2, (b) $Ti_{0.95}Fe_{0.05}O_{1.97}$, (c) $Ti_{0.90}Fe_{0.10}O_{1.95}$, (d) $Ti_{0.85}Fe_{0.15}O_{1.92}$, (e) $Ti_{0.80}Fe_{0.20}O_{1.90}$, (f) $Ti_{0.70}Fe_{0.30}O_{1.85}$, (g) $Ti_{0.60}Fe_{0.40}O_{1.80}$, (h) $Ti_{0.50}Fe_{0.50}O_{1.75}$. Anatase and rutile TiO_2 polymorphs and Ti_5O_9 phases were used as models for the refinements. The phases employed for each refinement are shown at the top of the plots.

Table S5: PDF real space Rietveld refinement parameters of $Ti_{1-x}Fe_xO_{2-0.5x}$ (*i.e.*, $0 \leq x \leq 0.5$) employing a combination of anatase + rutile + Ti_5O_9 or rutile + Ti_5O_9 as model.

* Short- to medium-range order real space Rietveld refinements primarily reflect local structural order rather than the full nanoparticle size, thus mean particle size diameter

Sample	Rw	Phase model	Scale factor	δ2 (Å^2)	a lattice parameter (Å)	b lattice parameter (Å)	c lattice parameter (Å)	ADP$_{Ti}$ (Å^2)	ADP$_{Fe}$ (Å^2)	ADP$_{O}$ (Å^2)
$Ti_{0.80}Fe_{0.20}O_{1.90}$	0.15	Anatase	0.08	2.40	3.7894	3.7894	9.4837	0.0047	0.0057	0.0348
		Rutile	0.46	2.08	4.5948	4.5948	2.9593	0.0072	0.0074	0.0203
		Ti_5O_9	0.11	0.91	5.4248	7.1930	8.9576	0.0061	0.0067	0.0032
$Ti_{0.70}Fe_{0.30}O_{1.85}$	0.22	Rutile	0.38	2.98	4.5922	4.5922	2.9582	0.0081	0.0050	0.0194
		Ti_5O_9	0.37	0.29	5.4408	7.2109	8.7601	0.0074	0.0343	0.0075
$Ti_{0.60}Fe_{0.40}O_{1.80}$	0.26	Rutile	0.28	2.45	4.5904	4.5904	2.9595	0.0068	0.0068	0.0207
		Ti_5O_9	0.52	2.29	5.4278	7.1905	8.8085	0.0107	0.0279	0.0199
$Ti_{0.50}Fe_{0.50}O_{1.75}$	0.28	Rutile	0.25	2.44	4.5873	4.5873	2.9594	0.0069	0.0068	0.0224
		Ti_5O_9	0.70	2.38	5.4261	7.2132	8.7751	0.0691	0.0077	0.0234
$Ti_{0.50}Fe_{0.50}O_{1.75}$	0.43	Ti_5O_9	0.95	2.12	5.4838	7.2414	8.4307	0.0322	0.0045	0.0142

was not included in the refined parameters table.

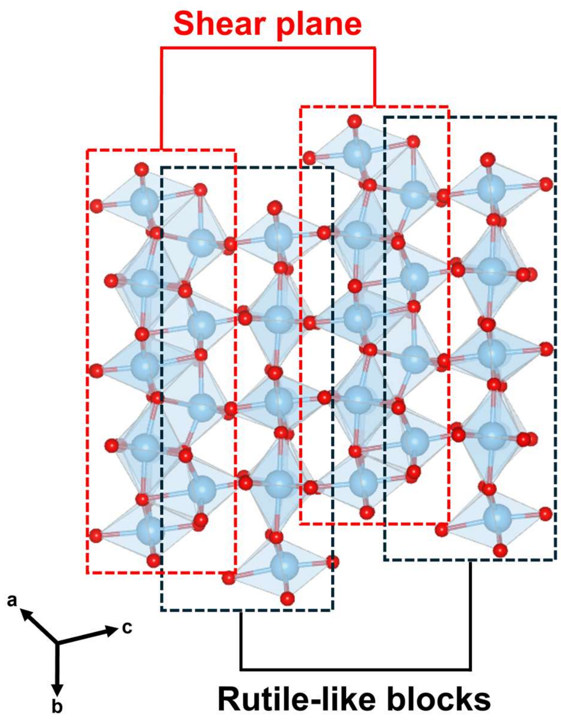

Figure S11: Structural representation of Ti_5O_9 (ICSD CollCode 653560) supercell (1x2x2) with highlighted regions in red representing shear planes and black rutile-like blocks.

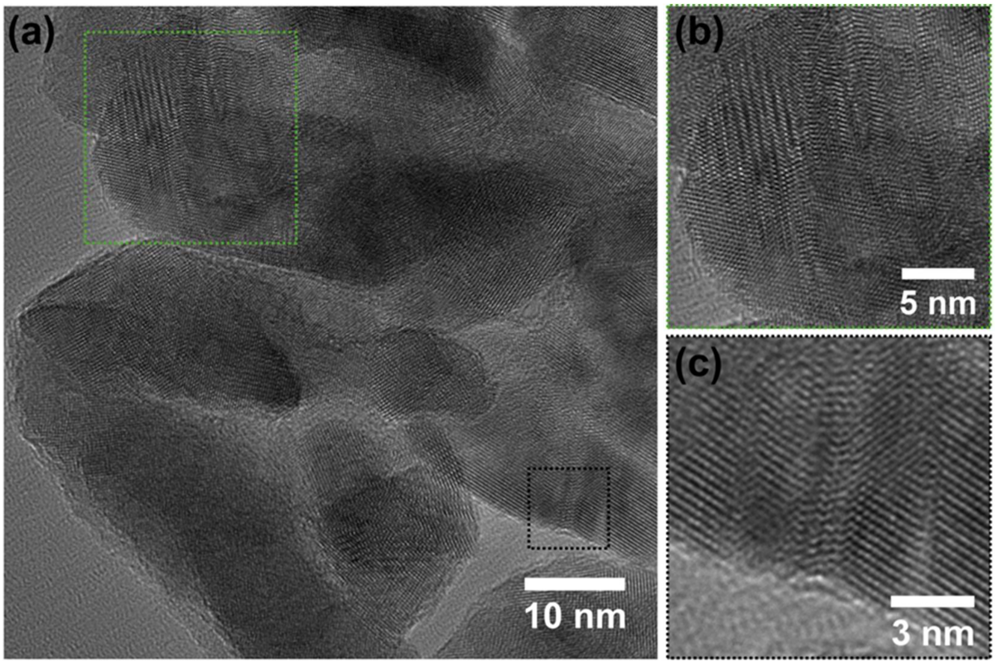

Figure S12: Representative HR-TEM image of (a) $Ti_{0.50}Fe_{0.50}O_{1.75}$ and highlighted defective regions (b) highlighted in green and (c) highlighted in black.

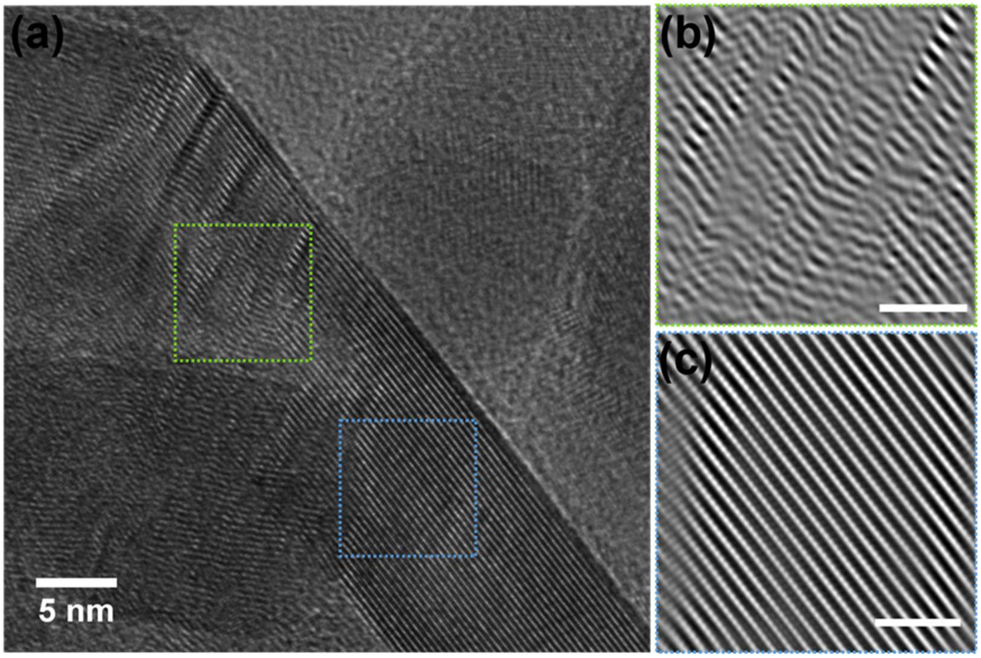

Figure S13: Representative HR-TEM image of (a) $Ti_{0.70}Fe_{0.30}O_{1.85}$ and IFFT of the (b) defective region (highlighted in green) and (c) non-defective region (highlighted in blue). Scale bar for IFFT images of 2 nm.

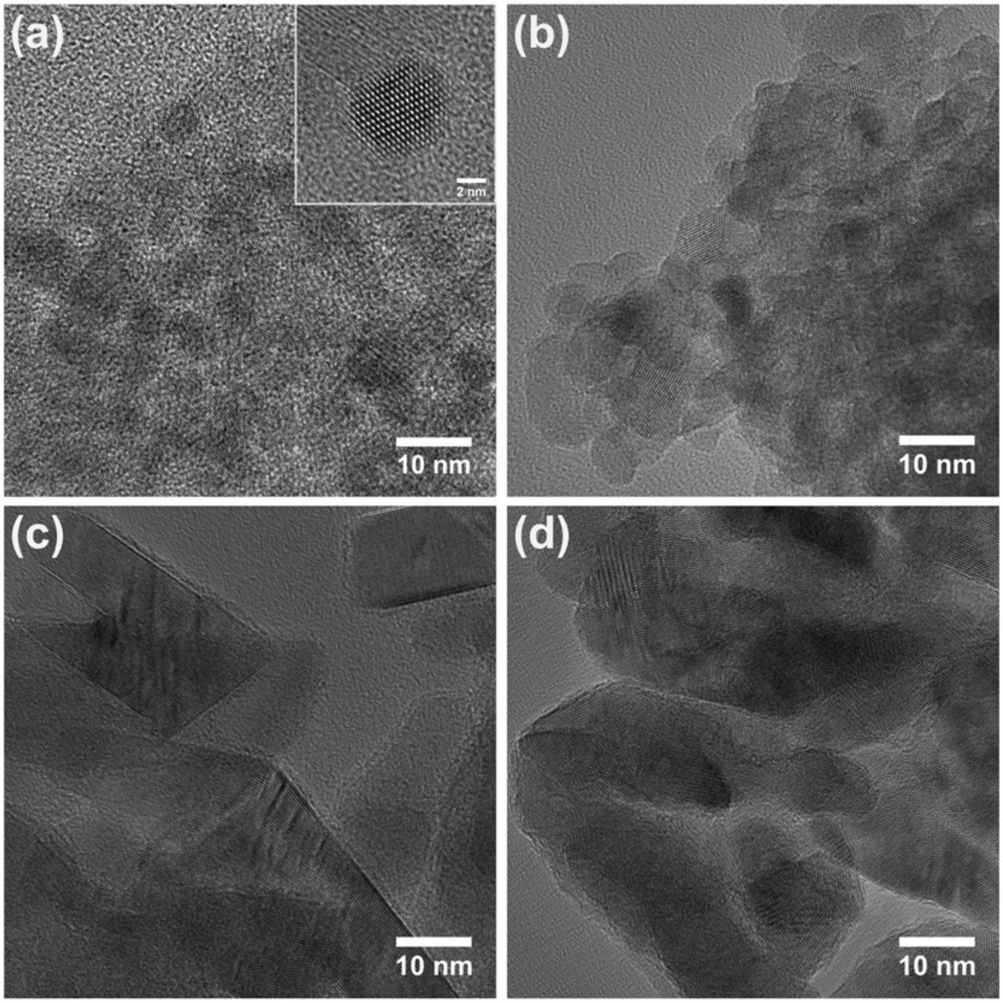

Figure S14: Representative HR-TEM images of (a) TiO_2 (inset: HR-TEM image of a TiO_2 nanoparticle), (b) $Ti_{0.90}Fe_{0.10}O_{1.95}$, (c) $Ti_{0.70}Fe_{0.30}O_{1.85}$, and (d) $Ti_{0.50}Fe_{0.50}O_{1.75}$.

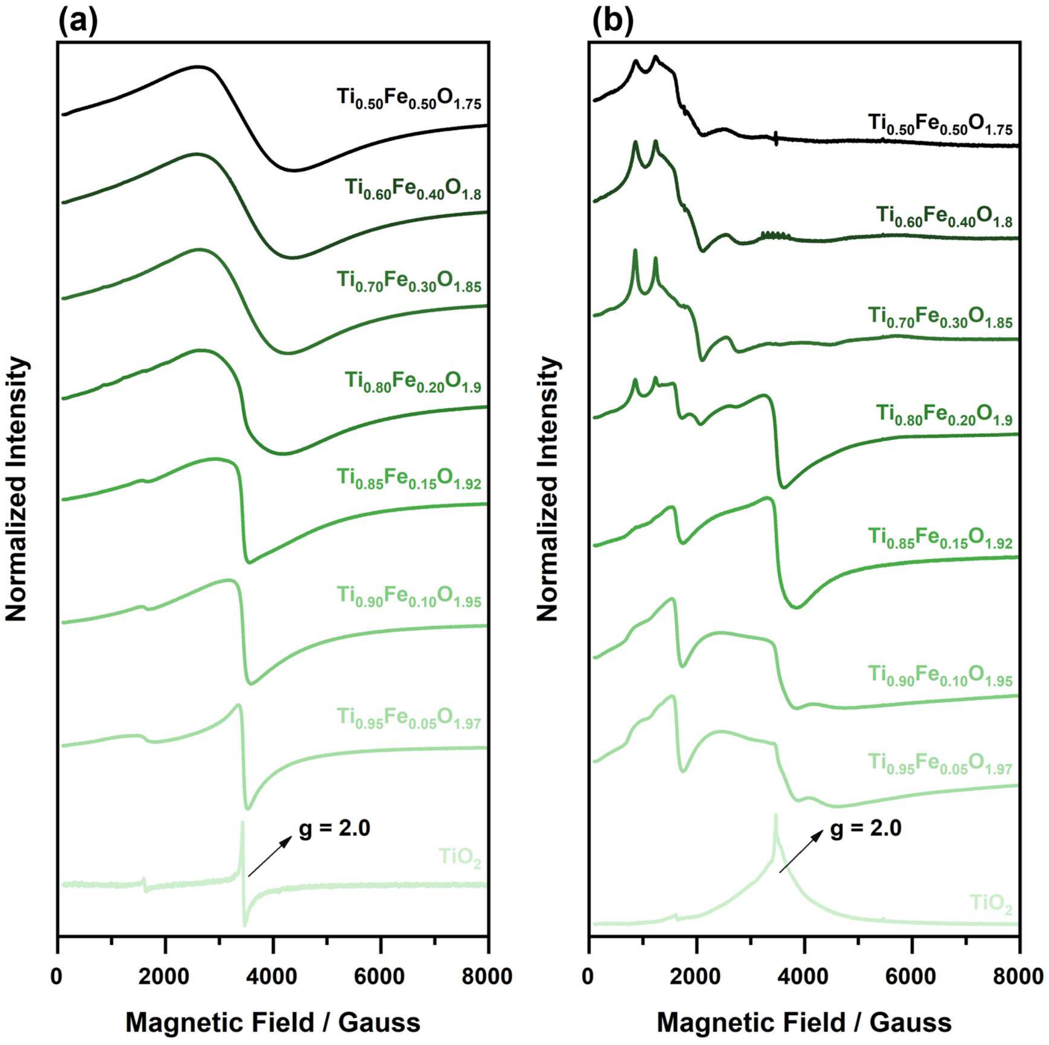

Figure S15: (a) Room temperature and (b) low temperature (5K) EPR measurements of $Ti_{1-x}Fe_xO_{2-0.5x}$ nanostructures as a function of the Fe loading ($0 \leq x \leq 0.5$).

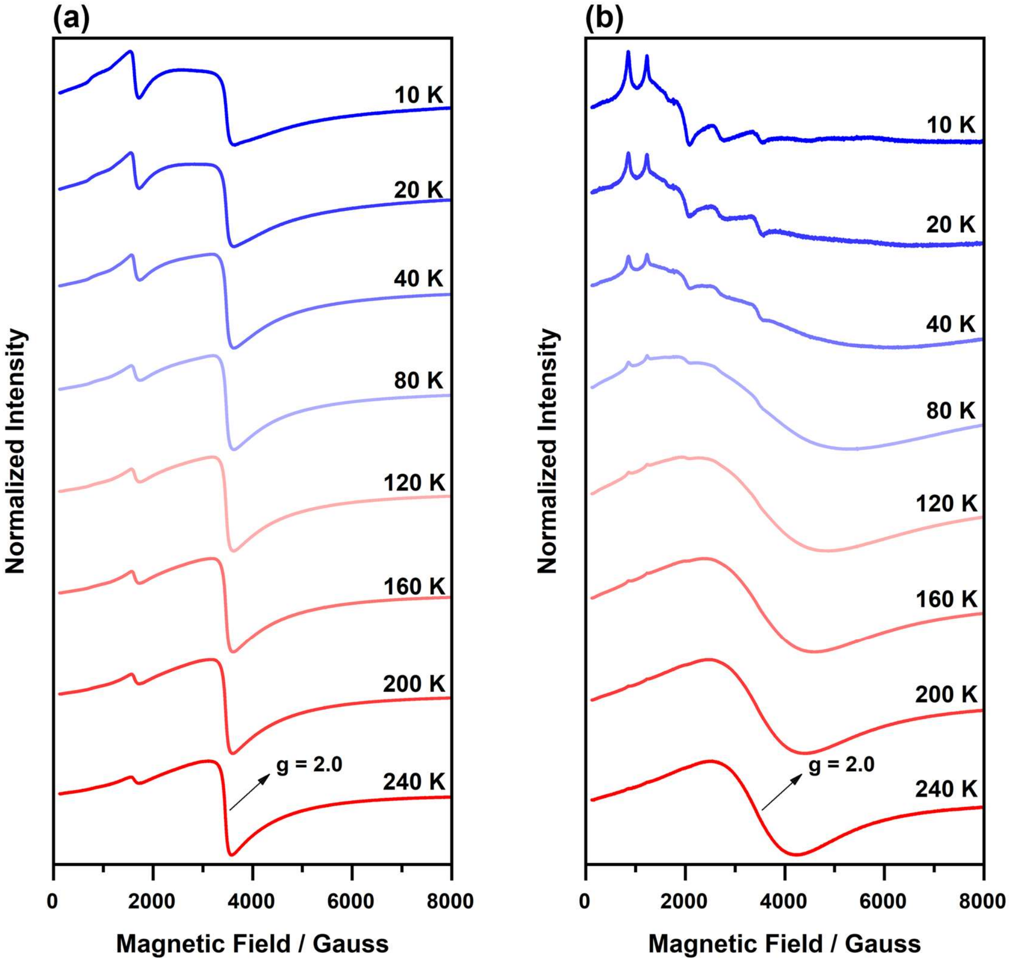

Figure S16: Temperature-dependent EPR measurements of (a) $Ti_{0.90}Fe_{0.10}O_{1.95}$ and (b) $Ti_{0.70}Fe_{0.30}O_{1.85}$. The temperature of each spectrum is shown in the graphs.

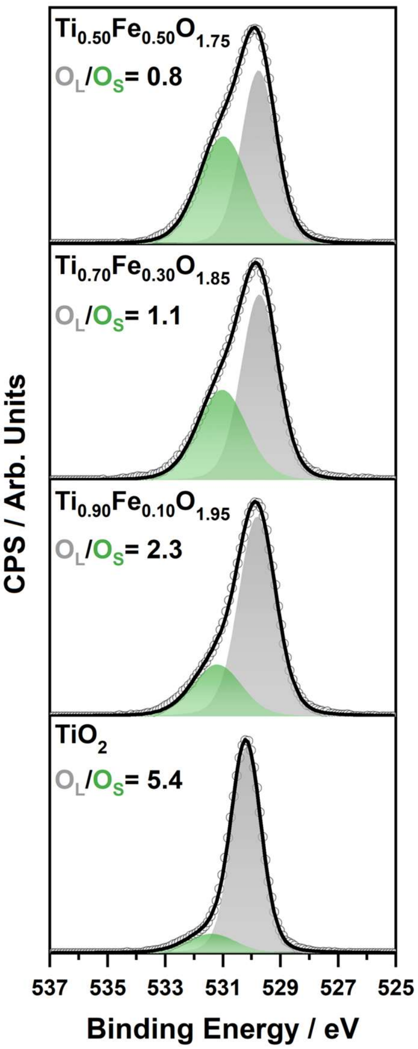

Figure S17: O 1s core spectra of $Ti_{1-x}Fe_xO_{2-0.5x}$, with x = 0, 0.1, 0.3, and 0.5. The gray contribution represents oxygen species in the oxide lattice, whereas the green contribution represents adsorbed oxygen species on the surface of the oxide. The ratios of lattice oxygen (O_L) and adsorbed oxygen (O_S) are presented within the graph.

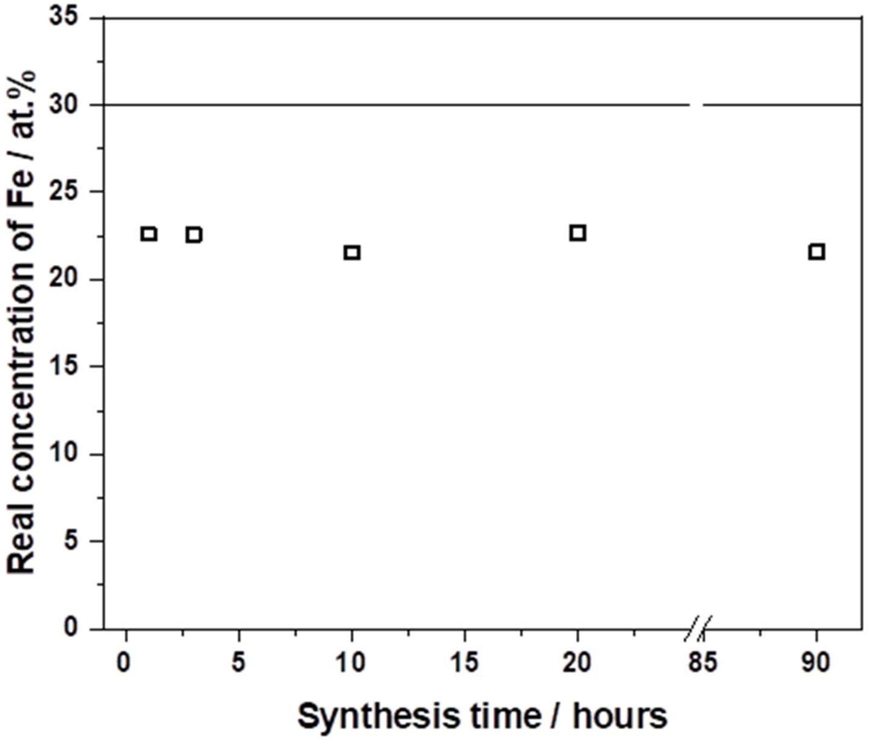

Figure S18: Correlation of the nominal (black line) and real Fe (at. %) concentrations (open squares) measured by ICP-OES of the $Ti_{0.70}Fe_{0.30}O_{1.85}$ sample as a function of synthesis time.

Table S6: Fe (at.%) concentrations of the $Ti_{0.70}Fe_{0.30}O_{1.85}$ sample as a function of synthesis time, measured by ICP-OES (nominal value = 30 at.%)

**Sample**	**Concentration of Fe / at.%**
$Ti_{0.70}Fe_{0.30}O_{1.85}$_1h	22.6
$Ti_{0.70}Fe_{0.30}O_{1.85}$_3h	22.6
$Ti_{0.70}Fe_{0.30}O_{1.85}$_10h	21.6
$Ti_{0.70}Fe_{0.30}O_{1.85}$_20h	22.7
$Ti_{0.70}Fe_{0.30}O_{1.85}$_90h	21.6

Table S7: Anatase Eg (ca. 150 cm^{-1}) and rutile Eg (ca. 440 cm^{-1}) center and FWHM as a function of synthesis time.

Time (hours)	Anatase Eg center (cm^{-1})	Anatase Eg FWHM (cm^{-1})	Rutile Eg center (cm^{-1})	Rutile Eg FWHM (cm^{-1})
1	155.1	32.1	-	-
3	154.1	26.5	-	-
10	150.5	-	437.3	58.2
20	-	-	437.0	58.7
90	-	-	438.8	58.4

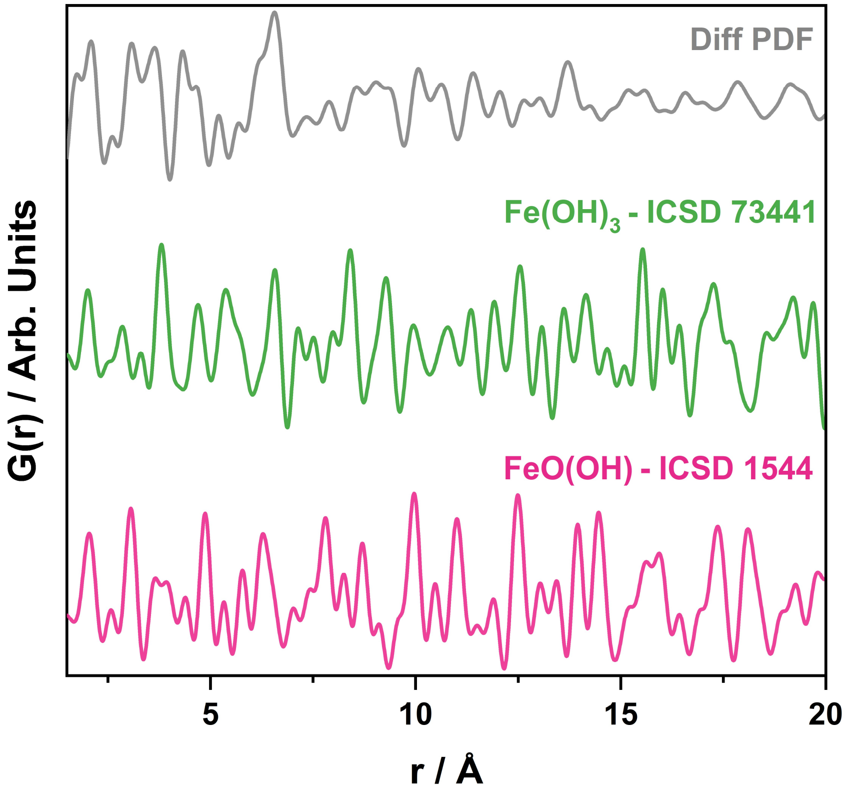

Figure S19: Residual PDF of $Ti_{0.70}Fe_{0.30}O_{1.85}$ when refined against an anatase phase model and simulated PDFs of $Fe(OH)_3$ and FeO(OH).

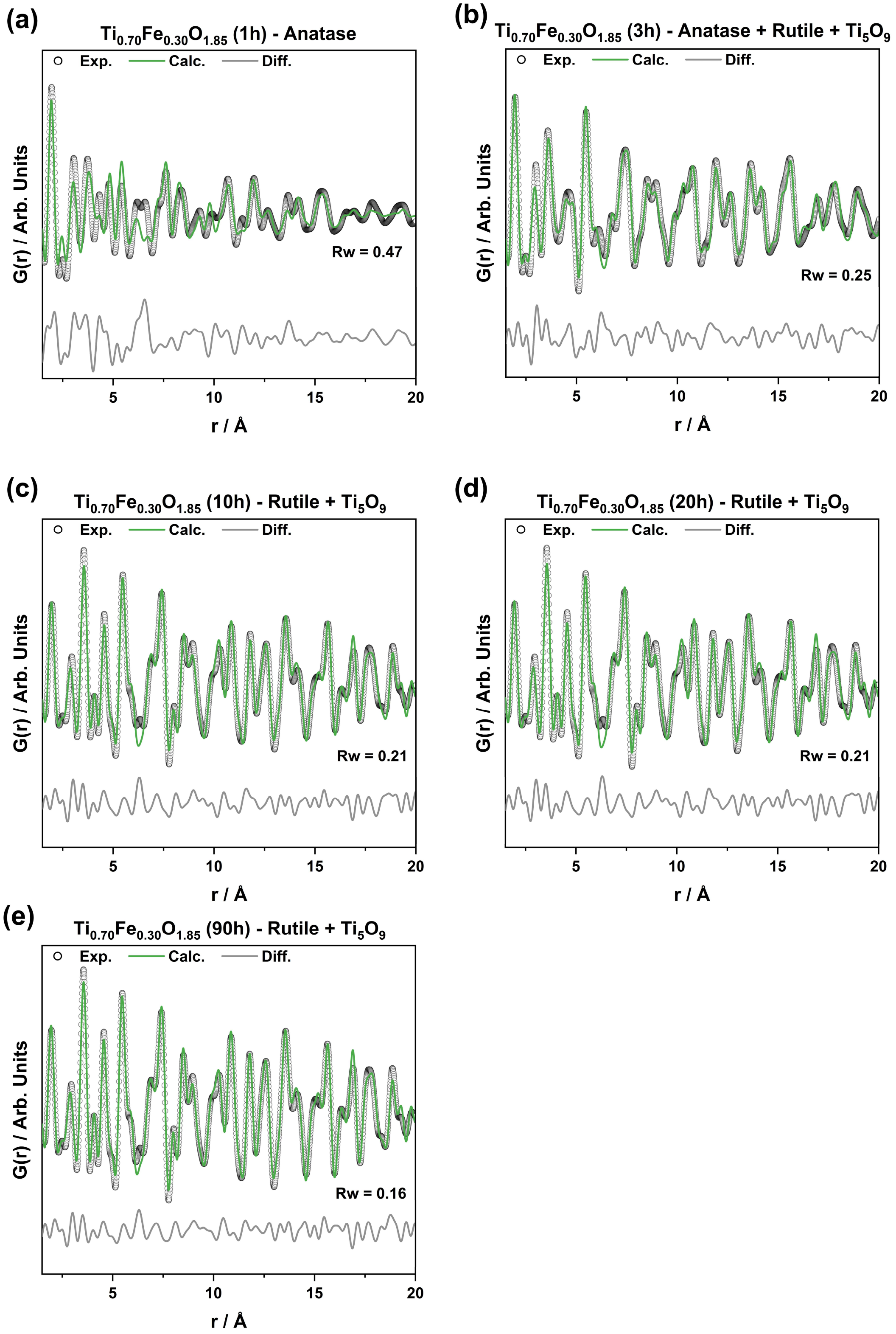

Figure S20: Short- to medium-range PDF refinements from *ex situ* measurements of $Ti_{0.70}Fe_{0.30}O_{1.85}$ as a function of synthesis time: (a) 1 h, (b) 3 h, (c) 10 h, (d) 20 h, (e) 90 h. The phases employed for each refinement are shown at the top of the plots.

Table S8: PDF real space Rietveld refinement parameters of $Ti_{0.70}Fe_{0.30}O_{1.85}$ as a function of synthesis time employing anatase or a combination of anatase + rutile + Ti_5O_9 or rutile + Ti_5O_9 as models.

Sample	Rw	Phase model	Scale factor	δ2 (Å^2)	a lattice parameter (Å)	b lattice parameter (Å)	c lattice parameter (Å)	ADP_{Ti} (Å^2)	ADP_{Fe} (Å^2)	ADP_O (Å^2)
$Ti_{0.70}Fe_{0.30}O_{1.85}$ – 1h	0.47	Anatase	0.48	3.65	3.7842	3.7842	9.5133	0.0090	0.0088	0.1984
$Ti_{0.70}Fe_{0.30}O_{1.85}$ – 3h	0.25	Anatase	0.19	1.40	3.7948	3.7948	9.4811	0.0030	0.0074	0.0391
		Rutile	0.14	3.64	4.5783	4.5783	2.9647	0.0064	0.0086	0.0664
		Ti_5O_9	0.51	2.90	5.4278	7.2330	8.7814	0.0103	0.2091	0.0341
$Ti_{0.70}Fe_{0.30}O_{1.85}$ – 10h	0.21	Rutile	0.32	2.66	4.5903	4.5903	2.9564	0.0050	0.0045	0.0169
		Ti_5O_9	0.40	1.24	5.3973	7.2481	8.6632	0.0068	0.0591	0.0249
$Ti_{0.70}Fe_{0.30}O_{1.85}$ – 20h	0.21	Rutile	0.33	2.89	4.5916	4.5916	2.9565	0.0051	0.0042	0.0165
		Ti_5O_9	0.41	0.47	5.3976	7.2498	8.6559	0.0067	0.0585	0.0227
$Ti_{0.70}Fe_{0.30}O_{1.85}$ – 90h	0.16	Rutile	0.37	2.49	4.5913	4.5913	2.9568	0.0054	0.0031	0.0141
		Ti_5O_9	0.38	1.22	5.4198	7.2546	8.5048	0.0055	0.0949	0.0397

* Short- to medium-range order real space Rietveld refinements primarily reflect local structural order rather than the full nanoparticle size, thus mean particle size diameter was not included in the refined parameters table.

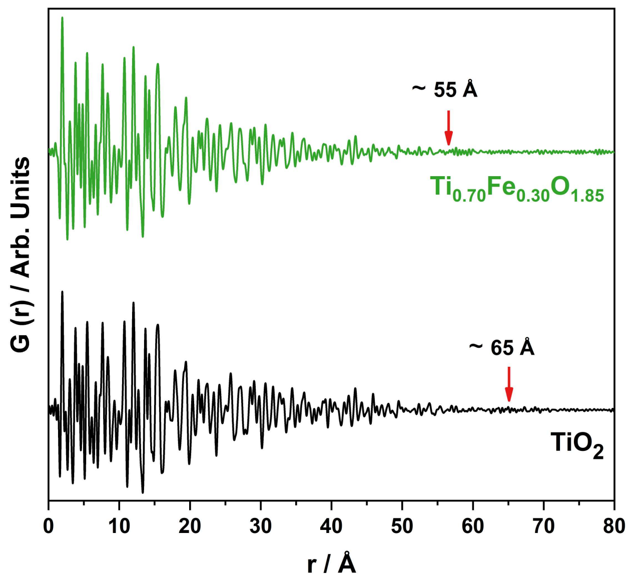

Figure S21: *In situ* hydrothermal short- to long-range order experimental PDFs of TiO_2 and $Ti_{0.70}Fe_{0.30}O_{1.85}$ after 20 min. Red arrows show the approximated correlation length observed in the experimental data.

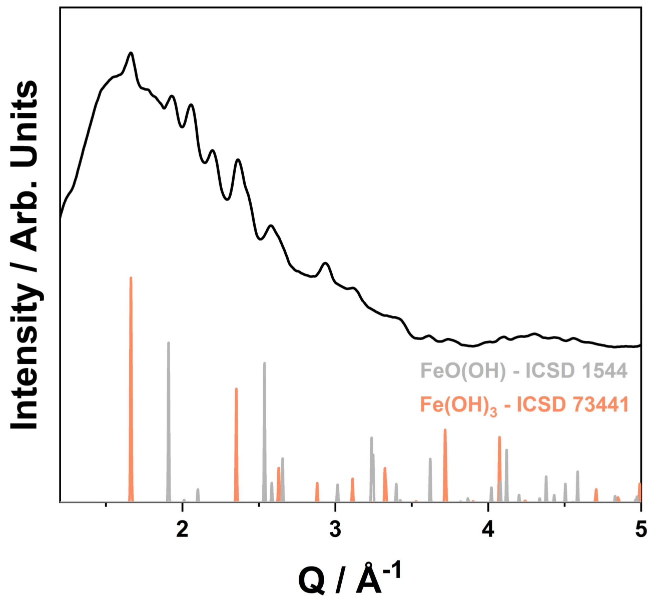

Figure S22: Integrated X-ray TS of $Ti_{0.70}Fe_{0.30}O_{1.85}$ at room temperature obtained from the experimental *in situ* hydrothermal TS data.

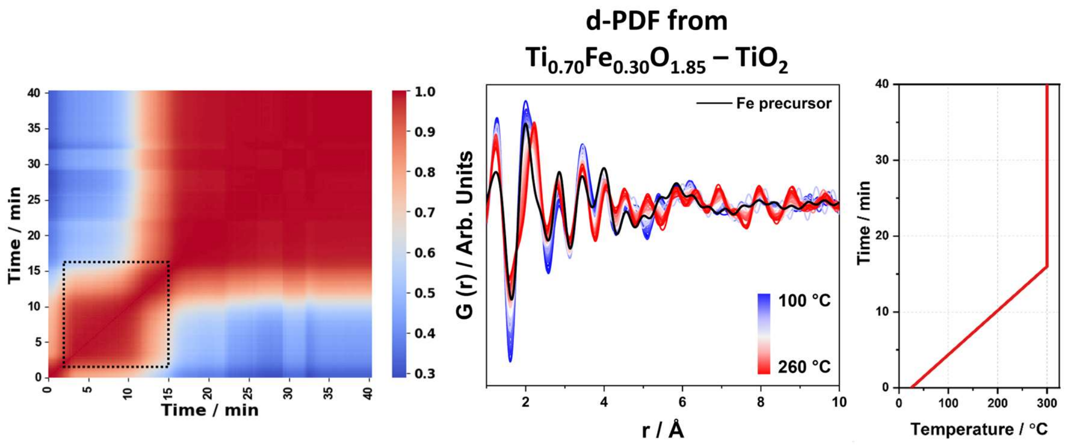

Figure S23: Comparison of Pearson correlation analysis of $Ti_{0.70}Fe_{0.30}O_{1.85}$ (left graph) and d-PDF of $Ti_{0.70}Fe_{0.30}O_{1.85} – TiO_2$ (middle graph, obtained at the same region as the one highlighted by the dashed square in the Pearson correlation plot), highlighting the formation of an intermediate as a function of temperature (right graph present the heating profile as a function of time). Fe precursor PDF stands for iron oxalate precursor in solution.

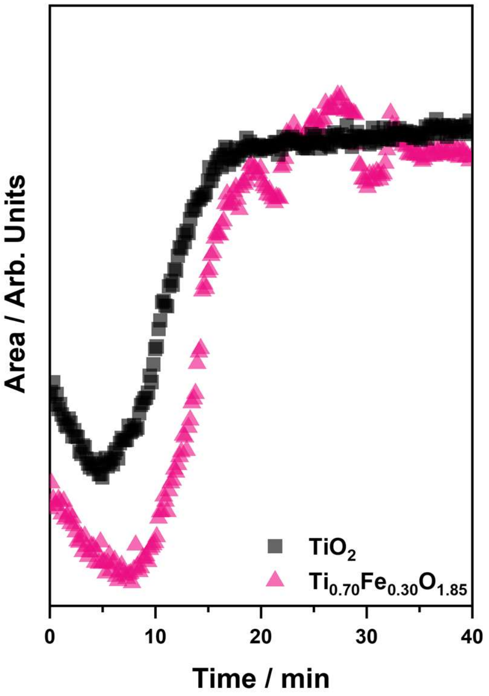

Figure S24: Integrated area of the experimental *in situ* PDF of TiO_2, $Ti_{0.90}Fe_{0.10}O_{1.95}$, and $Ti_{0.70}Fe_{0.30}O_{1.85}$ as a function of time, using the metal-metal distance associated with corner-sharing octahedra (*ca.* 3.8 Å).

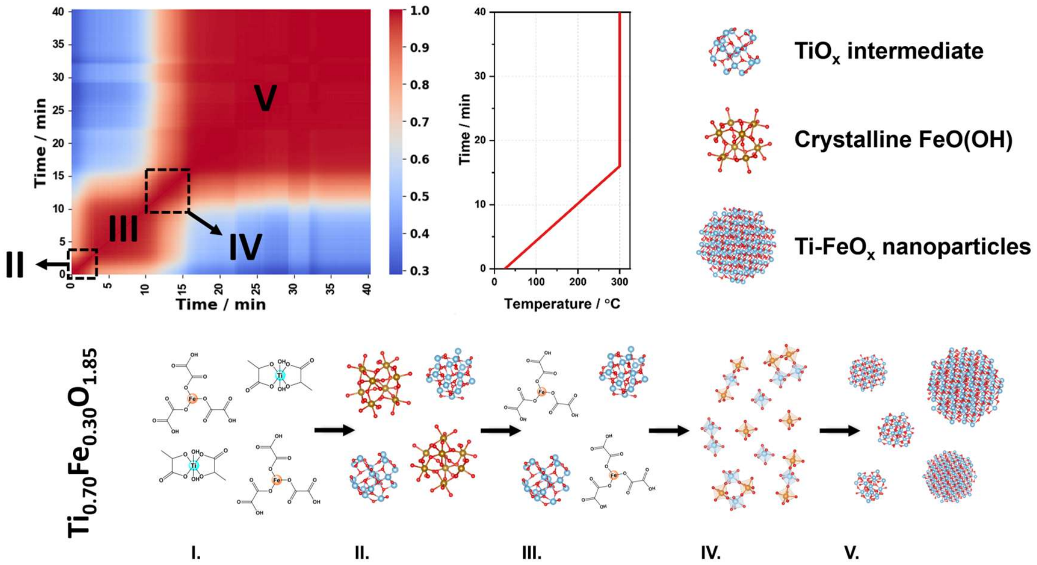

Figure S25: Correlation of synthesis events observed by the Pearson correlation analysis of the *in situ* hydrothermal PDF data of $Ti_{0.70}Fe_{0.30}O_{1.85}$ and the proposed synthesis mechanisms.

REFERENCES

(1) Kjær, E. T. S.; Aalling-Frederiksen, O.; Yang, L.; Thomas, N. K.; Juelsholt, M.; Billinge, S. J. L.; Jensen, K. M. Ø. In Situ Studies of the Formation of Tungsten and Niobium Oxide Nanoparticles: Towards Automated Analysis of Reaction Pathways from PDF Analysis Using the Pearson Correlation Coefficient. *Chemistry-Methods* 2022, *2* (9). https://doi.org/10.1002/cmtd.202200034.
